# Intermedin_1-53_ attenuates aging-associated vascular calcification in rats by upregulating sirtuin 1

**DOI:** 10.18632/aging.102934

**Published:** 2020-03-31

**Authors:** Yao Chen, Lin-Shuang Zhang, Jin-Ling Ren, Ya-Rong Zhang, Ning Wu, Mo-Zhi Jia, Yan-Rong Yu, Zhong-Ping Ning, Chao-Shu Tang, Yong-Fen Qi

**Affiliations:** 1 Laboratory of Cardiovascular Bioactive Molecule, School of Basic Medical Sciences, Peking University, Beijing 100083, China; 2 Key Laboratory of Molecular Cardiovascular Science, Ministry of Education, Peking University Health Science Center, Beijing 100083, China; 3 Department of Pathogen Biology, School of Basic Medical Sciences, Peking University, Beijing 100083, China; 4 Department of Gynaecology and Obstetrics, Beijing Chao-Yang Hospital, Capital Medical University, Beijing 100020, China; 5 Shanghai University of Medicine and Health Sciences, Shanghai University of Medicine and Health Sciences Affiliated Zhoupu Hospital, Shanghai 201318, China; 6 Department of Physiology and Pathophysiology, School of Basic Medical Sciences, Peking University, Beijing 100083, China

**Keywords:** intermedin, sirt1, aging, vascular calcification, vascular smooth muscle cell

## Abstract

Vascular calcification is a common phenomenon in older adults. Intermedin (IMD) is a cardiovascular bioactive peptide inhibiting vascular calcification. In this study, we aimed to investigate whether IMD_1-53_ attenuates aging-associated vascular calcification. Vascular calcification was induced by vitamin D3 plus nicotine (VDN) in young and old rats. The calcification in aortas was more severe in old rats treated with VDN than young control rats, and IMD expression was lower. Exogenous administration of IMD_1-53_ significantly inhibited the calcium deposition in aortas and the osteogenic transdifferentiation of vascular smooth muscle cells (VSMCs) in VDN-treated old rats. Moreover, levels of aging-related p16, p21 and β-galactosidase were all greatly decreased by IMD_1-53_. These results were further confirmed in rat and human VSMCs *in vitro*. In addition, *IMD*-deficient mouse VSMCs showed senescence features coinciding with osteogenic transition as compared with wild-type mouse VSMCs. Mechanistically, IMD_1-53_ significantly increased the expression of the anti-aging factor sirtuin 1 (sirt1); the inhibitory effects of IMD_1-53_ on calcification and senescence were blocked by *sirt1* knockdown. Furthermore, preincubation with inhibitors of PI3K, AMPK or PKA efficiently blunted the upregulatory effect of IMD_1-53_ on sirt1. Consequently, IMD_1-53_ could attenuate aging-associated vascular calcification by upregulating sirt1 via activating PI3K/Akt, AMPK and cAMP/PKA signaling.

## INTRODUCTION

Vascular calcification, an age-related pathology, refers to deposits of calcium and phosphate crystals in the form of hydroxyapatite precipitates in the vessel wall. Vascular calcification is a common pathophysiological process and a prevalent complication of atherosclerosis, hypertension, diabetes and chronic kidney disease (CKD) and is aggravated with aging. Osteochondrogenic transdifferentiation of vascular smooth muscle cells (VSMCs) may trigger the onset and progression of aging-associated vascular calcification because senescent VSMCs show reduced levels of the markers smooth muscle-22 alpha (SM-22α) and alpha-smooth muscle actin (α-SMA) and increased expression of the osteogenic factors runt-related transcription factor 2 (RUNX2), bone morphogenetic protein 2 (BMP2) and alkaline phosphatase (ALP) [1–3].

Numerous studies have shown that risk factors accumulating during aging contribute to VSMC osteogenic differentiation and vascular calcification, such as increased levels of the cyclin-dependent kinase inhibitors p16 and p21, extracellular matrix remodeling-related collagen deposition and elastin degradation, impaired DNA and increased levels of prelamin A and reactive oxygen species, and matrix vesicles released by injured endothelium [2–5]. Recently, reduced levels of aging-related calcification inhibitors such as matrix γ-carboxyglutamic acid (Gla) protein (MGP), anti-aging and anti-calcification factor α-klotho and sirtuin 1 (sirt1), were found to greatly promote vascular calcification [6–10].

Sirt1 is a nicotinamide adenine dinucleotide (NAD^+^)-dependent deacetylase and regulates cell aging, energy metabolism, apoptosis, genomic stability, and stress responses by deacetylating histones and a number of non-histone proteins [11]. Sirt1 is a well-known longevity factor: it can extend the lifespan of yeast, *Caenorhabditis elegans* and flies and delay the aging process of mammals [12, 13]. Sirt1 is highly expressed in the vasculature and protects against age-related cardiovascular diseases, including cardiac remodeling [14, 15], atherosclerosis [16], abdominal aortic aneurysm [17], and vascular calcification [8, 9]. Recent study demonstrated that cultured aortas of mice with *sirt1* knockdown showed accelerated medial calcification induced by inorganic phosphate [18]. Moreover, sirt1 downregulation promoted VSMC senescence and calcification under osteogenic conditions; mechanistically, sirt1 retards senescence-related VSMC calcification by inhibiting the aging marker p21 and osteogenic transcription factor RUNX2 [8, 9], so sirt1 may play a pivotal role in aging-associated vascular calcification.

Many studies have shown that endogenous paracrine/autocrine factors are involved in vascular calcification [7, 19, 20]. Intermedin (IMD), also known as adrenomedullin 2 (ADM2), is a secreted peptide that belongs to the calcitonin gene-related peptide (CGRP) superfamily and was discovered in 2004 [21, 22]. Human IMD gene encodes a prepropeptide of 148 amino acids with a signal peptide for secretion at the N terminus. IMD_1-53_ can be generated from prepro-IMD by proteolytic cleavage at Arg93-Arg94, which may be the main active fragment of IMD [23, 24]. IMD exerts its biological effects by non-selectively binding to the calcitonin receptor-like receptor (CRLR) and receptor activity modifying protein 1 (RAMP1), 2 and 3. Our previous research showed that exogenous IMD_1–53_ may attenuate CKD-associated vascular calcification by upregulating α-klotho and vitamin D3 plus nicotine (VDN)-induced vascular calcification by increasing MGP in young rats [6, 7]. In addition, IMD_1–53_ treatment could improve vascular function by increasing endothelial nitric oxide synthase activity [25] and inhibiting reactive oxygen species production [26], which may affect vascular aging [4]. However, whether IMD inhibits aging-associated vascular calcification is unclear.

Recent studies found that some cardiovascular bioactive peptides could regulate the aging process via activation of sirt1 [20, 27]. In this study, we investigated whether IMD has a regulatory effect on sirt1 and thus exerts protective effects on aging-associated vascular calcification.

## RESULTS

### IMD and its receptor levels in aging-associated vascular calcification induced by VDN in rats

**Figure 1:**
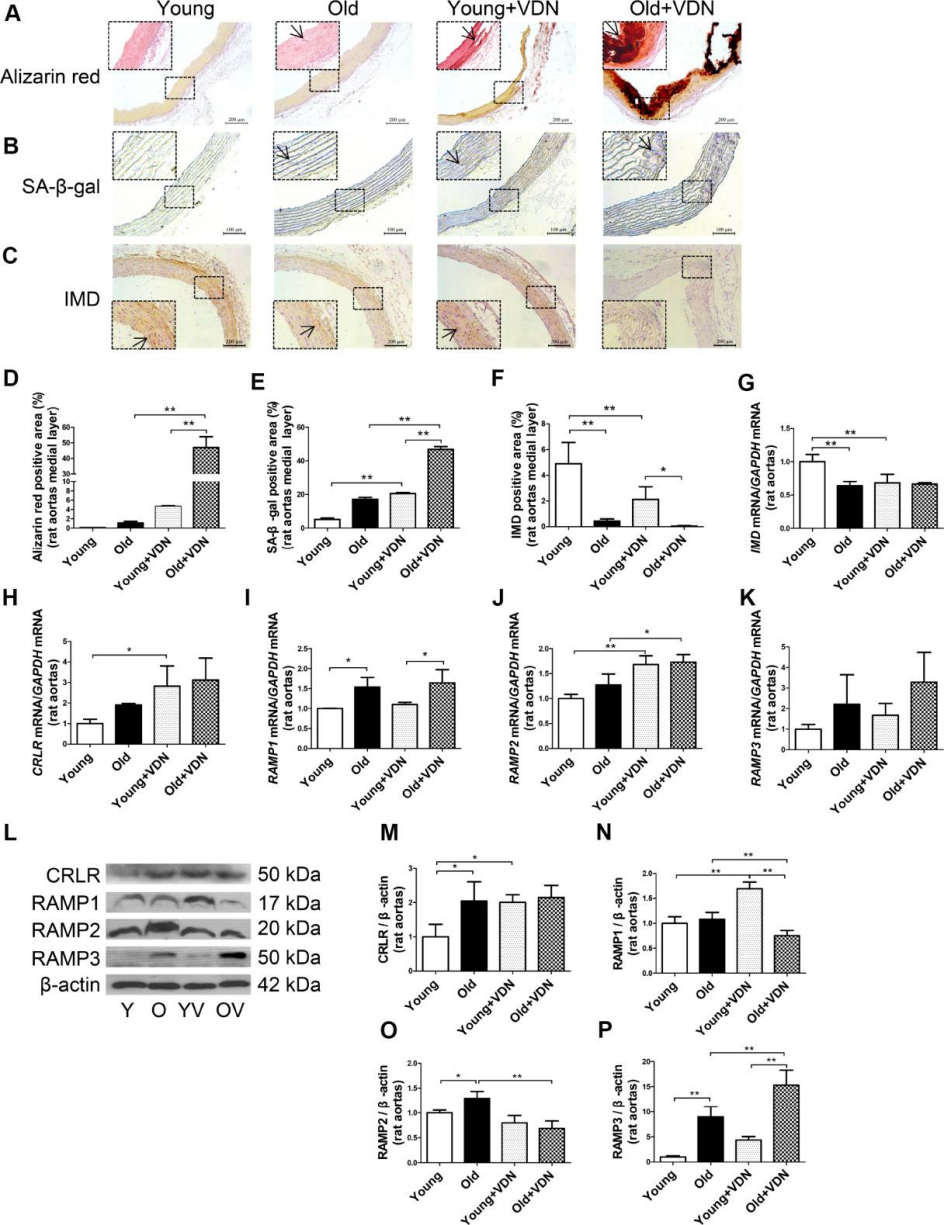
**IMD and its receptor levels in aging-associated vascular calcification induced by VDN in rats.** (**A**) Alizarin red staining for vascular calcium deposition (positive staining: red) (Scale bar=200 μm). (**B**) SA-β-gal staining for β-galactosidase activity (blue) (Scale bar=100 μm). (**C**) Immunohistochemistry staining for IMD (Scale bar=200 μm), and (**D**–**F**) quantification of (**D**) calcium deposition-positive staining (n=3), (**E**) β-galactosidase-positive staining (n=3) and (**F**) IMD-positive staining (n=4) in the medial layer of rat thoracic aortas. (**G**–**K**) Quantitative RT-PCR analysis of mRNA levels of *IMD*, calcitonin receptor-like receptor (*CRLR*), receptor activity-modifying protein 1 (*RAMP1*), *RAMP2* and *RAMP3* in rat aortas (n=3 in each group). (**L**) Western blot analysis of protein levels of CRLR and RAMP1, 2 and 3 in rat aortas and (**M**–**P**) quantification (n=3). The arrow indicates positive staining. Y=young rats. O=old rats. YV=young+VDN. OV=old+VDN. Data are mean ± SD. *P<0.05, **P<0.01.

First, we assessed vascular calcification and aging features in rats. As compared with controls, VDN-treated old rats with calcification showed substantially increased calcium deposition and senescence-associated β-galactosidase activity in the aortic media, as revealed by Alizarin red staining (Figure 1A, 1D) and SA-β-gal staining (Figure 1B, 1E).

We investigated whether IMD level was altered in the progression of aging-associated vascular calcification. On immunohistochemistry, the protein expression of IMD was significantly decreased by 91.2% (*P*<0.01) in old rat aortas as compared with young rats and further decreased in calcified old rat aortas (Figure 1C, 1F). *IMD* mRNA expression was lower by 36.0% (*P*<0.01) in old than young aortas, with no further decline in calcified old aortas (Figure 1G). The mRNA levels of IMD receptors *CRLR* and *RAMP1*, *2* and *3* were increased in calcified aortas of young or old rats versus non-calcified aortas, respectively (Figure 1H–1K). We then tested the protein expression of CRLR/RAMPs and found only RAMP3 protein level elevated in VDN-treated old aortas, by 3.5-fold (*P*<0.01) as compared with VDN-treated young aortas, and by 1.7-fold (*P*<0.01) as compared with old control aortas (Figure 1L–1P). We tested *IMD* expression in rat replicative senescent VSMCs (passage 14-18) and found decreased expression of *IMD* mRNA, and that of its receptors, CRLR/RAMP2/3 proteins, was increased, as compared with young VSMCs (passage 3-6) (Supplementary Figure 1A–1E).

### Exogenous IMD_1-53_ attenuated aging-associated vascular and VSMC calcification

**Figure 2:**
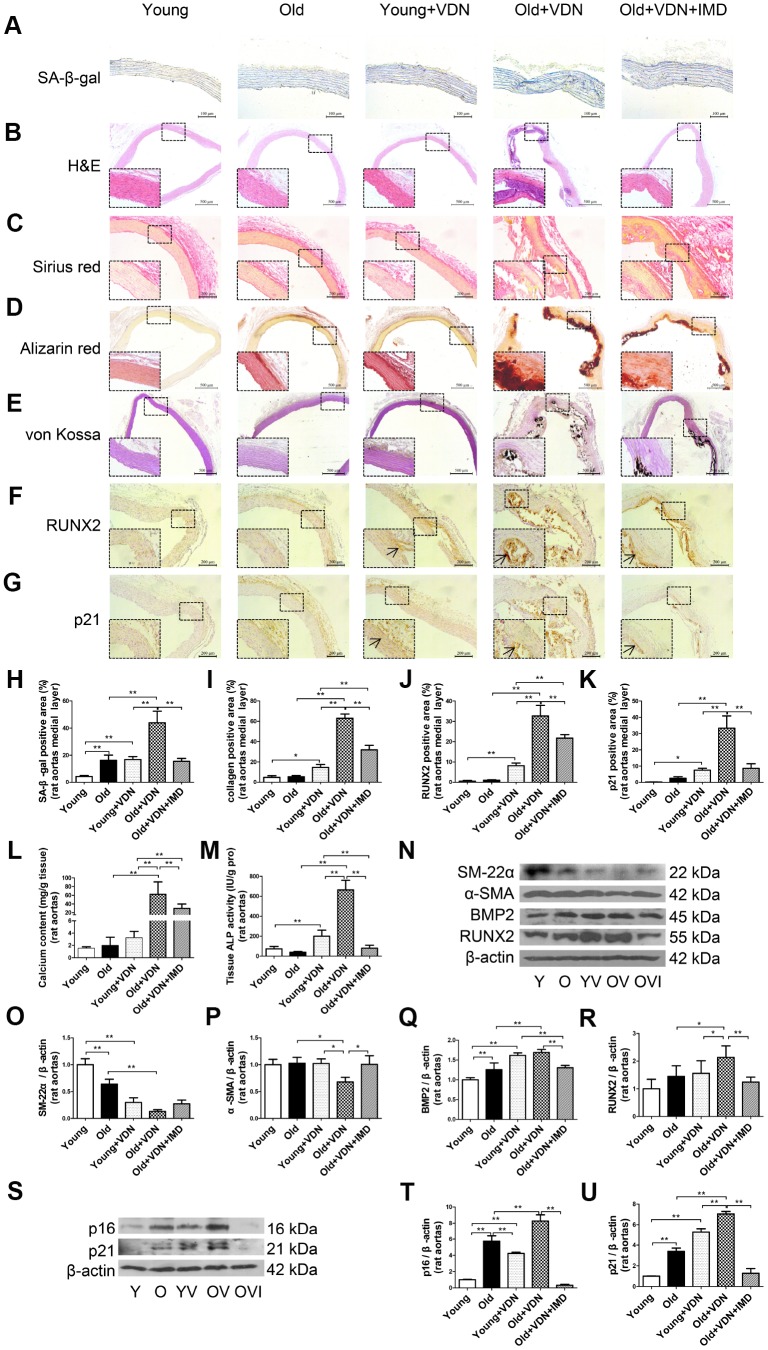
**Exogenous IMD_1-53_ attenuated aging-associated vascular calcification in rats.** (**A**) SA-β-gal staining for β-galactosidase activity (positive staining: blue) (Scale bar=100 μm), (**B**) H&E staining (Scale bar=500 μm), (**C**) Sirius red staining for collagen (red) (Scale bar=200 μm), (**D**) Alizarin red staining (red) (Scale bar=500 μm) and (**E**) von Kossa staining for vascular calcium deposition (black) (Scale bar=500 μm), (**F**) Immunohistochemistry staining for runt-related transcription factor 2 (RUNX2) (Scale bar=200 μm) and (**G**) cyclin-dependent kinase inhibitor p21 (Scale bar=200 μm), and (**H**–**K**) quantification of (H) β-galactosidase-positive staining (n=4), (**I**) collagen-positive staining (n=4), (**J**) RUNX2-positive staining (n=4), and (**K**) p21-positive staining (n=4) in the medial layer of rat thoracic aortas. (**L**) Calcium content assay (n=6) and (**M**) alkaline phosphatase (ALP) activity assay (n=6) in rat aortas. (**N**) Western blot analysis of protein levels of smooth muscle 22 alpha (SM-22α), alpha smooth muscle actin (α-SMA), bone morphogenetic protein 2 (BMP2) and RUNX2 in rat aortas, and (**O**–**R**) quantification (n=3). (**S**) Western blot analysis of protein levels of p16 and p21 in rat aortas, and (**T**, **U**) quantification (n=3). Enlarged regions, ×400. The arrow indicates positive staining. Y=young rats; O=old rats; YV=young+VDN; OV=old+VDN; OVI=old+VDN+IMD_1-53_. Data are mean ± SD. *P<0.05, **P<0.01.

To investigate whether exogenous IMD_1-53_ could attenuate aging-associated vascular calcification, we further administrated IMD_1-53_ in VDN-treated old rats. As compared with young rat aortas, the β-galactosidase activity was markedly upregulated in old aortas, and further increased in VDN-treated old rats (Figure 2A, 2H). The vascular structure was severely damaged in VDN-treated old rats (Figure 2B), with the increased collagen content (Figure 2C, 2I) and calcium deposition (Figure 2D, 2E). While IMD_1-53_ administration significantly decreased β-galactosidase activity by 64.7% (*P*<0.01, Figure 2A, 2H) and decreased collagen content by 49.4% (*P*<0.01, Figure 2C, 2I) in the medial layer of calcified old rat aortas, as compared with VDN-treated old rats. IMD_1-53_ also inhibited the calcium accumulation in aortas (Figure 2D, 2E). In addition, IMD_1-53_ treatment slightly improved the haemodynamic parameters of VDN-treated old rats (Supplementary Table 2). Consistent with the results of calcium deposition staining (Figure 2D, 2E), the calcium content and ALP activity were also downregulated with IMD_1-53_ treatment, by 52.3% (*P*<0.01, Figure 2L) and 88.0% (*P*<0.01, Figure 2M), respectively, as compared with VDN-treated old aortas. The increased protein levels of pro-osteogenic markers including RUNX2 and BMP2 and reduced levels of VSMC contractile markers SM-22α and α-SMA in VDN-treated old rat aortas were all reversed by IMD_1-53_ (Figure 2N–2R). Consistently, immunohistochemistry staining showed that IMD_1-53_ decreased the expression of RUNX2 in the aortic media layer with calcification (Figure 2F, 2J). Moreover, the protein levels of cyclin-dependent kinase inhibitors p16 and p21 were significantly upregulated in VDN-treated rats, then markedly reduced by 96.0% and 81.9% with IMD_1-53_ treatment (both *P*<0.01), respectively, as compared with VDN-treated old rats (Figure 2S–2U). Immunohistochemistry staining also showed that IMD_1-53_ decreased the expression of p21 in medial aorta cystic areas (Figure 2G, 2K).

**Figure 3:**
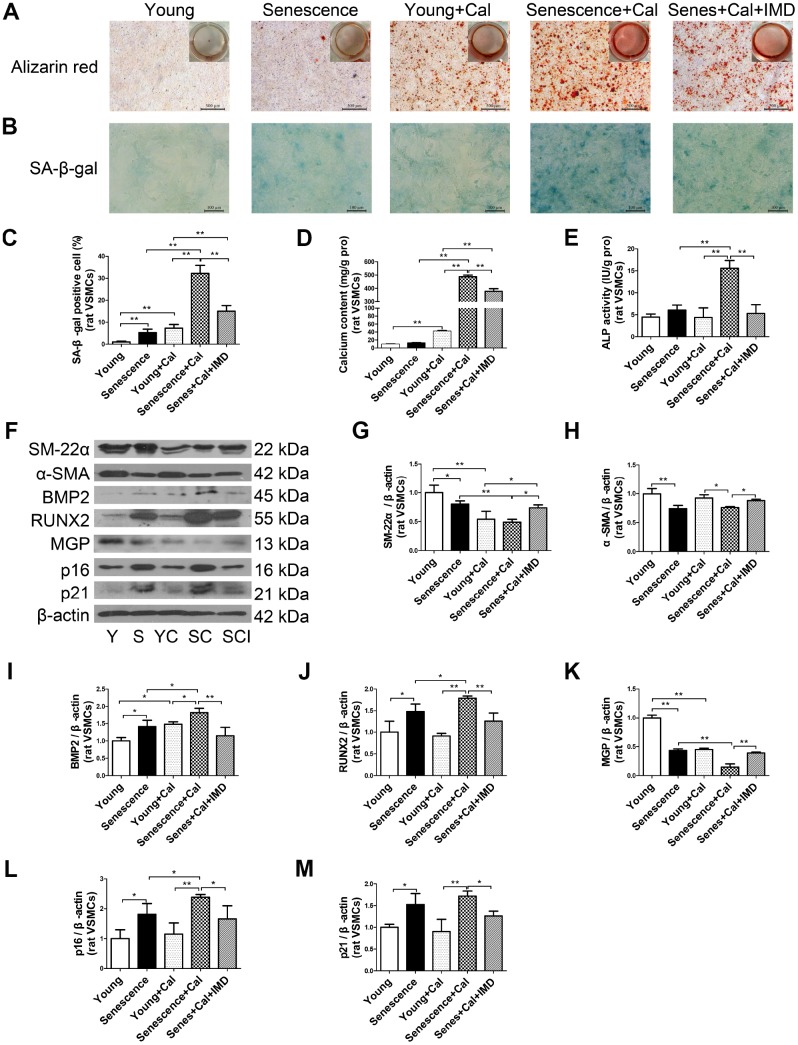
**IMD_1-53_ inhibited senescence-associated calcification in rat VSMCs.** (**A**) Alizarin red staining for rat VSMCs (positive staining: red) (Scale bar=500 μm). (**B**) SA-β-gal staining (blue) (Scale bar=100 μm) and (**C**) quantification of β-galactosidase-positive staining in rat VSMCs (n=6). (**D**) Calcium content assay (n=6) and (**E**) ALP activity assay (n=6) of rat VSMCs. (**F**) Western blot analysis of protein levels of smooth muscle 22 alpha (SM-22α), alpha smooth muscle actin (α-SMA), bone morphogenetic protein 2 (BMP2), runt-related transcription factor 2 (RUNX2), matrix γ-carboxyglutamic acid (Gla) protein (MGP), and cyclin-dependent kinase inhibitors p16 and p21 in rat VSMCs, and (**G**–**M**) quantification (n=3). Y=young VSMCs; S=senescent VSMCs; YC=young+calcification; SC=senescence+calcification; SCI=senescence+calcification+IMD_1-53_. Data are mean ± SD. *P<0.05, **P<0.01.

Given the importance of VSMC senescence and phenotype transition in aging-associated vascular calcification, we tested the effects of IMD_1-53_ on VSMC *in vitro*. Alizarin red staining showed earlier and more severe calcification in senescent VSMCs than young cells (Supplementary Figure 2A). Consistent with *in vivo* experiments, the calcification markers including calcium deposition and content (Figure 3A, 3D), ALP activity (Figure 3E), and protein levels of RUNX2 and BMP2 (Figure 3F, 3I, 3J) were elevated in calcified-rat senescent VSMCs as compared with young controls, but α-SMA and SM-22α levels were significantly decreased (Figure 3F–3H). Moreover, the protein level of MGP, an inhibitor of calcification, was also downregulated in calcified VSMCs (Figure 3F, 3K). The senescent markers β-galactosidase activity (Figure 3B, 3C) and the expression of p16 and p21 (Figure 3F, 3L, 3M) were markedly increased in calcified-rat senescent VSMCs. All these changes were reversed by treatment with IMD_1-53_ (all *P*<0.05, Figure 3A–3M).

The effect of IMD_1-53_ on senescent VSMC calcification was further confirmed in human VSMCs *in vitro* (Supplementary Figure 3A–3L).

Taken together, these results demonstrate that IMD_1-53_ could attenuate aging-associated vascular calcification by inhibiting VSMC osteogenic transition and senescence.

### Deficiency of *IMD* promoted senescence and calcification in VSMCs *in vitro*

**Figure 4:**
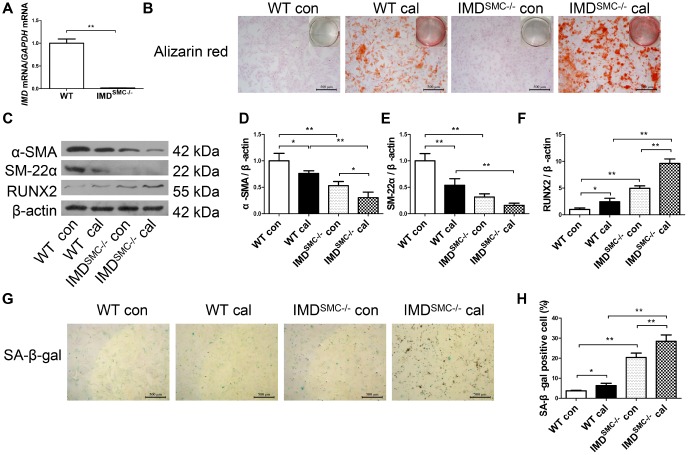
**Deficiency of *IMD* promoted senescence and calcification in VSMCs *in vitro.*** (**A**) RT-PCR analysis of mRNA level of *IMD* in VSMCs from WT and IMD^SMC-/-^ mice (n=3). (**B**) Alizarin red staining (positive staining: red) (scale bar=500 μm) of mice VSMCs (passage 5-6). (**C**) Western blot analysis of protein levels of α-SMA, SM-22α and RUNX2, and (**D**–**F**) quantification (n=3). (**G**) SA-β-gal staining (blue) (scale bar=500 μm) and (**H**) quantification of β-galactosidase-positive staining (n=6). WT=wild type. IMD^SMC-/-^=VSMC-specific *IMD*-deficient. Con=control. Cal= calcification. Data are mean ± SD. *P<0.05, **P<0.01.

To further confirm the role of IMD in senescence-associated calcification, VSMCs were isolated from wild type (WT) and VSMC-specific *IMD*-deficient (IMD^SMC-/-^) mice. RT-PCR verified that *IMD* was knocked out in IMD^SMC-/-^ VSMCs (Figure 4A). *IMD*-deficient VSMCs showed significantly accumulated calcium deposition as compared with WT VSMCs under the osteogenic condition (Figure 4B). The protein level of the VSMC osteogenic transition marker RUNX2 was upregulated and those of contractile markers α-SMA and SM-22α were downregulated in calcified *IMD*-deficient VSMCs as compared with calcified WT VSMCs, by 3.9-, 2.5- and 3.4-fold (all *P*<0.01), respectively (Figure 4C–4F). *IMD*-deficient VSMCs showed significantly increased β-galactosidase activity, by 5.5-fold (*P*<0.01), in control media as compared with WT VSMCs, which was further increased after induction of calcification (Figure 4G, 4H). Altogether, our data demonstrate that endogenous IMD was essential for protecting VSMCs against calcification and senescence.

### IMD_1-53_ inhibited aging-associated vascular calcification by increasing sirt1 expression and deacetylase activity

**Figure 5:**
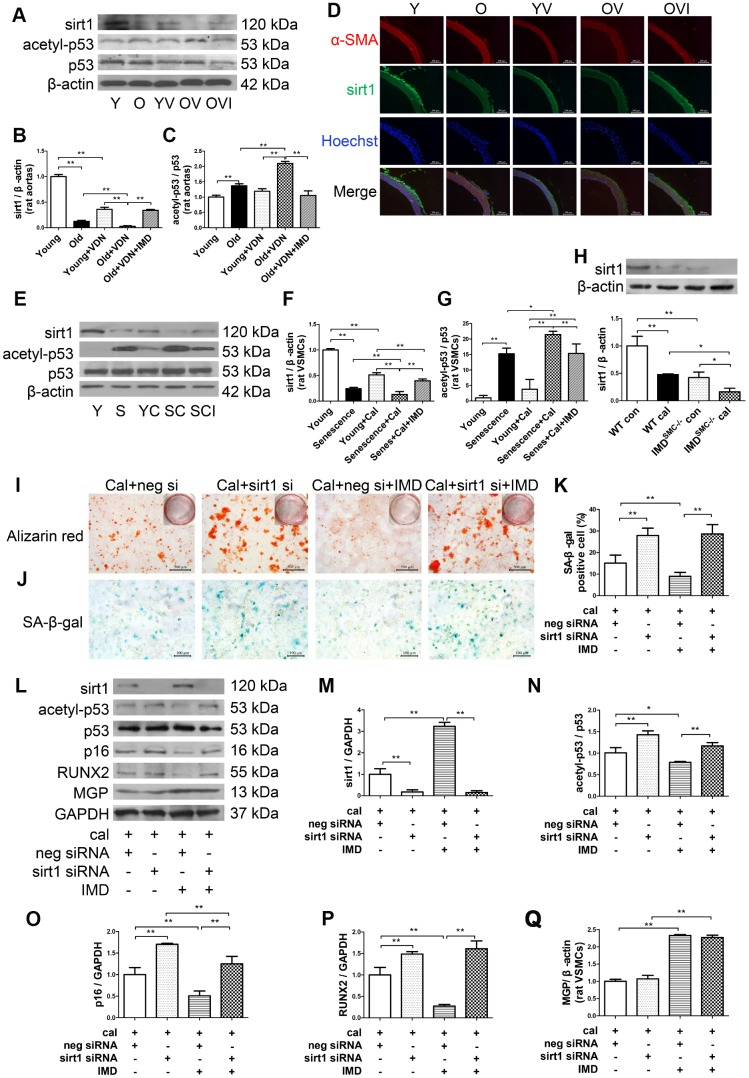
**IMD_1-53_ inhibited aging-associated vascular calcification by increasing sirt1 expression and deacetylase activity.** (**A**) Western blot analysis of protein levels of sirt1, acetylation p53 (acetyl-p53), and total p53 (p53) in rat aortas, and (**B**, **C**) quantification (n=3). (**D**) Immunofluorescence staining for α-SMA (red) and sirt1 (green) in rat aortas. Nuclei were stained with Hoechst 33342 (blue). Merged images (α-SMA, sirt1 and nuclei) are shown (Scale bar=200 μm). (**E**) Western blot analysis of protein levels of sirt1, acetyl-p53, and p53 in rat VSMCs and (**F**, **G**) quantification (n=3). (**H**) Western blot analysis and quantification of protein level of sirt1 in WT and IMD^SMC-/-^ mouse VSMCs (n=3). (**I**) Alizarin red staining (red) (Scale bar=500 μm) and (**J**) SA-β-gal staining (blue) (Scale bar=100 μm) of calcified-rat senescent VSMCs treated with IMD_1-53_ plus *sirt1* siRNA or negative siRNA, and (**K**) quantification of β-galactosidase-positive staining (n=6). (**L**) Western blot analysis of protein levels of sirt1, acetyl-p53, p53, p16, RUNX2 and MGP and (**M**–**Q**) quantification (n=3). For *in vivo* experiments, Y=young rats; O=old rats; YV=young+VDN; OV=old+VDN; OVI=old+VDN+IMD_1-53_. For *in vitro* experiments, Y=young VSMCs; S=senescent VSMCs; YC=young+calcification; SC=senescence+calcification; SCI=senescence+calcification+IMD_1-53_. WT=wild type. IMD^SMC-/-^=VSMC-specific *IMD*-deficient. Con=control. Cal= calcification. neg si=negative siRNA. sirt1 si=sirt1 siRNA. Cal=calcification. Data are mean ± SD. *P<0.05, **P<0.01.

Sirt1, the NAD^+^-dependent histone deacetylase, is a well-known anti-aging factor and a key inhibitor of vascular calcification [8, 9]. We investigated whether IMD_1-53_ could inhibit aging-associated vascular calcification by upregulating sirt1 expression and deacetylase activity. Sirt1 protein level was significantly decreased in young and old aortas of rats with calcification as compared with control young or old rat aortas, respectively (Figure 5A, 5B). Immunofluorescence staining revealed a similar pattern (Figure 5D). *In vitro*, sirt1 protein level in calcified-rat senescent VSMCs was increased at day 6 (Supplementary Figure 4A, 4B), and then significantly decreased at day 12 with calcification time as compared with control VSMCs (Supplementary Figure 4A, 4B, Figure 5E, 5F). Also, both the mRNA and protein levels of sirt1 were significantly lower in IMD^SMC-/-^ than WT VSMCs (Supplementary Figure 4C, Figure 5H). IMD_1-53_ treatment significantly reversed the reduced sirt1 protein level in calcified old aortas (Figure 5A, 5B). The reduced sirt1 protein expression in calcified-rat senescent VSMCs was also restored by IMD_1-53_ (Figure 5E, 5F).

p53 is one of the deacetylation substrates of sirt1, and the ratio of acetylated p53 (acetyl-p53) to total p53 (p53) is an index of sirt1 activity [28, 29]. The acetyl-p53/p53 ratio was significantly increased in both calcified-rat old aortas (Figure 5A, 5C) and senescent VSMCs (Figure 5E, 5G), which indicated downregulated deacetylase activity of sirt1. The increased ratio of acetyl-p53/p53 was reversed by IMD_1-53_ *in vivo* (Figure 5A, 5C) and *in vitro* (Figure 5E, 5G).

The effect of IMD_1-53_ on sirt1 and acetyl-p53/p53 was further confirmed in human VSMCs (Supplementary Figure 4D–4F).

To explore the role of sirt1 in IMD_1-53_ inhibiting aging-associated vascular calcification, we knocked down *sirt1* by siRNA. In rat senescent VSMCs, the mRNA and protein levels of sirt1 were greatly decreased with *sirt1*-siRNA (Supplementary Figure 4G–4I). Calcium deposition and β-galactosidase activity were reduced with IMD_1-53_ treatment in negative siRNA-treated calcified-rat senescent VSMCs, which was reversed with *sirt1* knockdown (Figure 5I–5K). The ratio of acetyl-p53/p53 and levels of RUNX2 and p16 were also decreased with IMD_1-53_ treatment in negative siRNA-treated calcified-rat senescent VSMCs but significantly increased with *sirt1* knockdown (Figure 5L–5P). However, *sirt1* knockdown had no effect on MGP protein level in calcified-senescent VSMCs (Figure 5L, 5Q). Taken together, these data indicate that IMD_1-53_ attenuated aging-associated vascular calcification by upregulating sirt1 expression and deacetylase activity.

### Involvement of signaling pathways in IMD_1-53_ upregulating sirt1 in senescence-associated VSMC calcification

**Figure 6:**
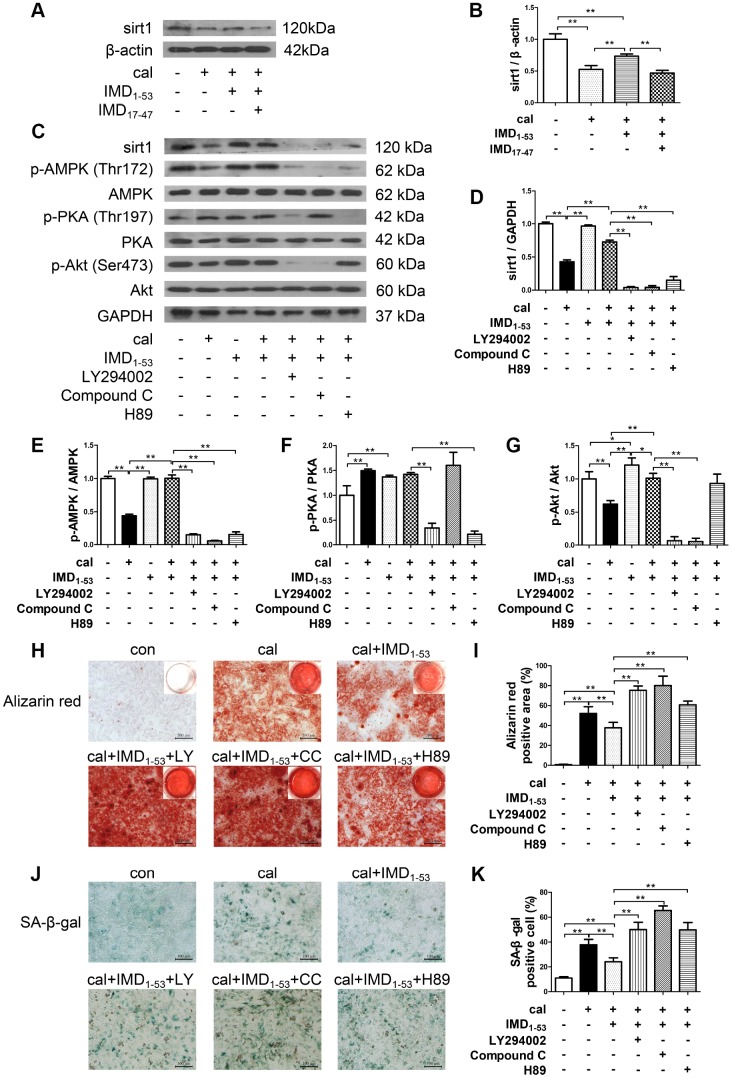
**Involvement of signaling pathways in IMD_1-53_ upregulating sirt1 in senescence-associated VSMC calcification.** (**A**) Western blot analysis of IMD_17-47_ (10^-6^ mol/L) blocking the effect of IMD_1–53_ on sirt1 protein level in calcified-rat senescent VSMCs, and (**B**) quantification (n=3). (**C**) Western blot analysis of protein levels of sirt1, phosphorylated-AMP-activated protein kinase (p-AMPK) (Thr172), AMPK, phosphorylated-protein kinase A (p-PKA) (Thr197), PKA, phosphorylated-protein kinase B (p-Akt) (Ser473), Akt in rat senescent VSMCs preincubation with or without phosphatidylinositol 3-kinase (PI3K) inhibitor LY294002, AMPK inhibitor Compound C or PKA inhibitor H89 (all 10 μmol/L) before IMD_1–53_ administration and calcification induction, and (**D**–**G**) quantification of (**D**) sirt1 (n=3), (**E**) p-AMPK/AMPK (n=3), (**F**) p-PKA/PKA (n=3), (**G**) p-Akt/Akt (n=3). (**H**) Alizarin red staining (Scale bar=200 μm), and (**I**) quantification of calcium deposition-positive staining (red) (n=6). (**J**) SA-β-gal staining (Scale bar=100 μm), and (**K**) quantification of β-galactosidase-positive staining (blue) (n=6). Con=control. Cal=calcification. Data are mean ± SD. *P<0.05, **P<0.01.

IMD exerts its biological effects mainly through signaling pathways downstream of its receptors CRLR/RAMPs [24, 26]. We further explored the role of CRLR/RAMPs in mediating the effect of IMD on sirt1 by pretreatment with IMD_17-47_, an antagonist of IMD receptor complex CRLR/RAMPs [21, 26]. The results showed that the upregulated protein expression of sirt1 by IMD_1-53_ in calcified-rat senescent VSMCs was blocked by IMD_17-47_, which suggested that IMD_1-53_ upregulates sirt1 by activating its receptor complex CRLR/RAMPs (Figure 6A, 6B).

We then investigated the signaling pathway by which IMD_1-53_ upregulates sirt1 in senescence-associated VSMC calcification. The results showed that preincubation with three kinase inhibitors, including phosphatidylinositol 3-kinase (PI3K) inhibitor LY294002, AMP-activated protein kinase (AMPK) inhibitor Compound C and protein kinase A (PKA) inhibitor H89, all blocked the upregulatory effect of IMD_1-53_ on sirt1 protein level (Figure 6C, 6D). It was showed that in calcified-rat senescent VSMCs, the level of AMPK and Akt phosphorylation was reduced as compared with control, which was reversed by IMD_1-53_ treatment. However, the level of PKA phosphorylation was elevated, which was unaffected by IMD_1-53_ treatment (Figure 6C, 6E–6G). In addition, in senescent VSMCs treated with IMD_1-53_ alone, both PKA and Akt were activated, but the p-AMPK level was unchanged, as compared with control (Figure 6C, 6E–6G). Furthermore, the inhibitory effects of IMD_1-53_ on VSMC calcification and senescence, as assessed by calcium deposition (Figure 6H, 6I) and β-galactosidase activity (Figure 6J, 6K), were blocked by LY294002, Compound C or H89 preincubation. Collectively, the anti-senescence-related VSMC calcification effects of IMD_1-53_ might be mediated by increasing sirt1 level via activating PI3K/Akt, AMPK and cAMP/PKA signaling pathways.

To investigate whether there is a relationship between klotho and sirt1 in IMD_1-53_ protecting against aging-associated vascular calcification, we examined the expression of klotho in rat VSMCs. As shown in Supplementary Figure 5A and 5B, klotho protein level significantly decreased in calcified-rat senescent VSMCs, which was reversed by IMD_1-53_. Preincubation with LY294002, Compound C or H89 all blocked the upregulatory effect of IMD_1-53_ on klotho level in calcified-rat senescent VSMCs (Supplementary Figure 5C, 5D). Moreover, sirt1 level was significantly reduced in the aortas of klotho^+/-^ mice, as compared to WT mice (Supplementary Figure 5E–5G). While the protein level of klotho remained unchanged after *sirt1* knockdown in *sirt1* siRNA-treated calcified-rat senescent VSMCs as compared with control (Supplementary Figure 5H, 5I). These results suggested that IMD_1-53_ may exert its protective role against aging-associated vascular calcification through a klotho-sirt1-axis via multiple pathways.

## DISCUSSION

**Figure 7:**
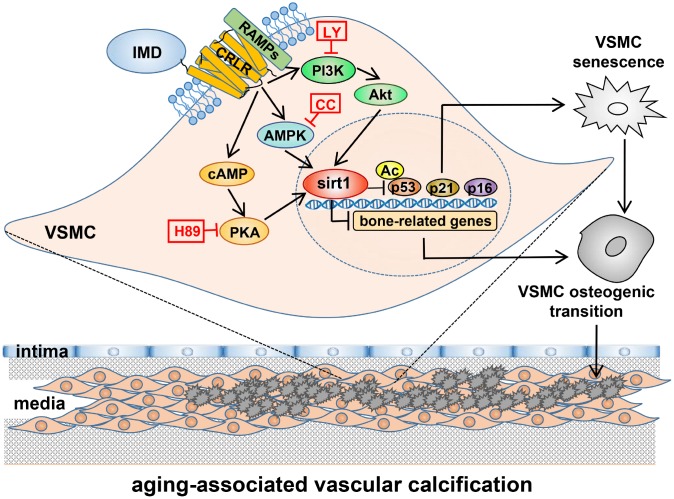
**A working model of mechanism through which IMD attenuates aging-associated vascular calcification.** IMD upregulates sirt1 expression and deacetylase activity via combining CRLR/RAMPs receptor complex and activating PI3K/Akt, AMPK and cAMP/PKA signaling. Upregulation of sirt1 by IMD inhibits VSMC osteogenic transdifferentiation and VSMC senescence, thus preventing the development of aging-associated vascular calcification. LY=LY294002, CC=Compound C.

In the present study, VDN-treated old rats showed severe vascular calcification as compared with young rats, accompanied by significantly decreased level of IMD and upregulated receptor complex CRLR/RAMP3. Exogenous administration of IMD_1-53_ significantly attenuated aging-associated calcification in VDN-treated old rat aortas or calcified-rat and human senescent VSMCs *in vitro*. Deficiency of endogenous *IMD* promoted senescence and calcification in VSMCs. Mechanistically, the level of the anti-aging factor sirt1 and deacetylase activity were significantly decreased in calcified old aortas and VSMCs, which was reversed by IMD_1-53_ treatment. The protective effects of IMD_1–53_ on senescence-related VSMC calcification were abolished with *sirt1* knockdown, and the upregulatory effect of IMD_1–53_ on sirt1 was blocked by inhibiting PI3K/Akt, AMPK or cAMP/PKA signaling. Taken together, IMD_1-53_ protected against aging-associated vascular calcification by upregulating sirt1 via activating PI3K/Akt, AMPK and cAMP/PKA signaling (Figure 7).

Aging is a vascular calcification factor. Arterial calcification occurs with aging in rats, but it is unobvious and its intensity is much lower than that observed in human [30, 31]. VDN induced rat vascular calcification model was first established in 1997 by Niederhoffer et al, which produced a vascular calcification intensity similar to that observed in the elderly, and was used to study aging-associated vascular calcification [30, 32]. In our study, calcium deposition in aortas was greater in VDN-treated old than young rats, and the treatment markedly upregulated levels of the bone-related genes RUNX2 and BMP2 and reduced that of the VSMC contractile markers SM-22α and α-SMA, thereby suggesting that aging promotes the development of calcification. Aging-associated β-galactosidase activity and levels of the cyclin-dependent kinase inhibitors p16 and p21 were significantly increased with VDN treatment, so calcification could in turn promote aging, which agrees with primary studies [3, 10].

The expression changes of IMD and its receptors varies by the state and stage of disease. Previous study reported that plasma IMD level was increased in acute myocardial infarction and acute coronary syndrome [33, 34] but downregulated in the aortic tissues of 5/6 nephrectomized-induced CKD rats and VDN-treated young rats [6, 7]. In our study, both mRNA and protein levels of IMD were reduced in calcified old rat aortas. We hypothesized that elevated level of IMD was a protective compensatory effect, and its downregulation might be closely related to an increase in disease severity. Like other CGRP members, IMD exerts its biological effects by combined activity of the co-receptors CRLR/RAMPs [24]. Similar to our previous study in CKD vascular calcification [7], we showed that CRLR/RAMP3 proteins were significantly upregulated in VDN-treated old rats. CRLR/RAMP3 may mediate the protection of IMD_1-53_ in aging-associated vascular calcification.

We found that IMD_1-53_ could attenuate aging-associated vascular calcification by directly inhibiting VSMC osteogenic transdifferentiation and by inhibiting VSMC senescence. VSMCs undergo an osteogenic phenotype change, and upregulate expression of bone-related factors could contribute to vascular calcification [35, 36]. Previously, we showed that IMD_1-53_ could increase the levels of VSMC contractile proteins and decrease those of osteogenic markers [6, 7]. In this study, we confirmed the inhibitory effects of IMD_1-53_ on VSMC osteogenic transdifferentiation, which directly inhibited aging-associated calcification. p16 and p21, which block cell cycle progression, are the most widely used biomarkers for aging [37, 38]. Expression of p16 and p21 in senescent VSMCs is usually accompanied by upregulation of bone-related genes such as ALP, RUNX2 and type I collagen [1, 3, 39], so VSMCs adopt a specific calcifying phenotype during senescence. Also, p16 and p21 are upregulated in calcified VSMCs, and knockdown of p21 could inhibit senescent-related VSMC calcification [8, 10]. We found that IMD_1-53_ significantly reduced the expression of p16 and p21 *in vivo* and *in vitro*, which suggested that IMD_1-53_ may also attenuate aging-associated vascular calcification by inhibiting VSMC senescence. We also found the *IMD*-deficient VSMCs showed significantly increased senescence-associated β-galactosidase activity along with increased RUNX2 level and decreased α-SMA and SM-22α protein levels as compared with WT VSMCs, which confirmed that IMD is a key inhibitor of aging-associated vascular calcification.

The mRNA and protein levels of sirt1 were lower in *IMD*-deficient than WT VSMCs, but exogenous IMD_1-53_ could attenuate the aging-associated vascular calcification by increasing sirt1 expression and deacetylase activity. Sirt1 is one of the most critical inhibitors of the aging process: it can reduce oxidative stress, increase NO production, induce autophagy, decrease inflammation, and prevent cell senescence [11, 40]. As well, these effects may result in inhibiting vascular calcification [4]. Recently, a few studies provided direct evidence that sirt1 ameliorates vascular calcification [8, 9, 18]. One study found that hyperphosphatemia-induced VSMC senescence and calcification was associated with downregulation of sirt1 [8]. Another study showed that miR-34a promoted vascular calcification via VSMC mineralization by inhibiting cell proliferation and inducing senescence with direct sirt1 downregulation [9]. Sirt1 exerts its function by deacetylation of histone or non-histone proteins [11]. p53 is one of the deacetylated substrates of sirt1, and the ratio of acetyl-p53 to total p53 indicates the activity of sirt1 [28, 29]. Sirt1 inhibits p53-dependent cell cycle arrest and apoptosis and promotes cell survival and proliferation [41]. Sirt1-mediated deacetylation of p53 could inhibit human VSMC calcification and arterial stiffness in mice [8, 28]. Similarly, we found that calcification induced a significant increase in acetyl-p53 level in old rat aortas and replicative senescent rat and human VSMCs. The ratio of acetyl-p53/p53 was completely reversed by IMD_1-53_ treatment. Our results suggest that IMD upregulates the mRNA and protein expression and deacetylase activity of sirt1 and indicate that sirt1-mediated p53 deacetylation plays an important role in the anti-aging-associated vascular calcification effects of IMD_1-53_.

PI3K/Akt, AMPK and cAMP/PKA have been reported as downstream signaling pathways of IMD receptors [24]. IMD_1-53_ mediates a variety of biological effects such as inhibits cell proliferation, migration and apoptosis and protects against myocardial injury, abdominal aortic aneurysm, and vascular calcification by activating PI3K/Akt, AMPK or cAMP/PKA [7, 42, 43]. We showed that in calcified-senescent VSMCs, the levels of p-AMPK and p-Akt were downregulated as compared with control, but the level of p-PKA was upregulated. According to the previous studies, the activation of PKA during aging-associated calcification may be a compensatory protective effect [44, 45]. Here, we found that inhibitors of all these three signaling factors — PI3K inhibitor LY294002, AMPK inhibitor Compound C and PKA inhibitor H89 — efficiently blocked the upregulation of sirt1 by IMD_1-53_ and thus inhibited the effects of IMD_1-53_ on calcium deposition and β-galactosidase activity. In support of our findings, previous studies have reported that the three kinase inhibitors could inhibit sirt1 expression under other treatments [46–48]. Therefore, IMD_1-53_ may upregulate sirt1 by activating PI3K/Akt, AMPK and cAMP/PKA signaling during the development of aging-associated vascular calcification. However, in our study, the protein level of p-AMPK was positively correlated with the expression of sirt1, which was not found in p-Akt and p-PKA. Our results suggested that AMPK may be involved in the direct regulation of sirt1 during aging-associated vascular calcification. What remains to be fully elucidated is the relationship among these kinases, and their direct and indirect effects on sirt1 activation. Our previous study showed that IMD_1-53_ could upregulate klotho by activating cAMP/PKA pathway [7]. In the present study, IMD_1-53_ upregulated klotho protein expression in calcified-rat senescent VSMCs, which was inhibited by the use of the inhibitors of PI3K/Akt, AMPK or cAMP/PKA signaling. Consistent with previously [28], we showed that sirt1 protein level was decreased in *klotho*-deficiency mouse aortas. While the protein level of klotho remained unchanged after *sirt1* knockdown. Therefore, IMD_1-53_ may exert its protective role against aging-associated vascular calcification through a klotho-sirt1-axis via multiple pathways.

Further experimentation is required to explore the detailed mechanisms of how IMD_1-53_ regulates sirt1. Also, future works need to be studied in old IMD^SMC-/-^ mice.

In summary, we provide evidence that the paracrine/endocrine factor IMD has a protective role in aging-associated vascular calcification, and sirt1 is an important target for IMD. Therefore, strategies to maintain a high level of IMD may provide novel therapeutic opportunities for preventing vascular calcification during aging.

## MATERIALS AND METHODS

### Ethics Statement

All animal care and experimental protocols complied with the Guide for the Care and Use of Laboratory Animals published by the US National Institutes of Health (NIH Publication, 8^th^ Edition, 2011) and were approved by the Animal Care Committee of Peking University Health Science Center.

### Animals

We purchased 2-month old (young) and 16-month old (old) male Sprague-Dawley (SD) rats from Chengdu Dashuo Biological Technology Co. (Chengdu, China). IMD^SMC-/-^ mice were from Animal Research Center of Nanjing University (Nanjing, China) and were previously generated by crossing mice carrying loxP-flanked *IMD* alleles with SM22α-Cre transgenic mice. Klotho^+/-^ mice were from Animal Research Center of Nanjing University (Nanjing, China) were previously generated by crossing mice carrying loxP-flanked *klotho* allele with EIIa-Cre transgenic mice.

Rats were randomly assigned to 5 groups for treatment (n=10-12 each group): young (Y), old (O), young+VDN (YV), old+VDN (OV), and old+VDN+IMD_1-53_ (OVI). Vascular calcification was induced by VDN as described [6, 30, 49] with minor modification. Briefly, rats were given vitamin D3 (300,000 IU/kg in arachis oil, intramuscularly) simultaneously with nicotine (25 mg/kg in 5 mL peanut oil, intragastrically) at 9:00 on day 1. The nicotine administration was repeated at 18:00. At 14 days later, rats were re-treated with vitamin D3. The control group received normal saline intramuscularly and peanut oil without nicotine (5 mL/kg). IMD_1-53_ was administered subcutaneously (100 ng/kg/h, 4 weeks) (Phoenix Pharmaceuticals, Belmont, CA, USA) in phosphate buffered saline on day 2 via Alzet Mini-osmotic Pump (Alzetw model 2004, DURECT Corp., Cupertino, CA, USA) [6, 7]. On day 28 after VDN treatment, rats were anesthetized by intraperitoneal injection of pentobarbital (40 mg/kg), then underwent hemodynamic measurement with use of the Powerlab BL-420F Biological System (Tai-Meng Biotechnological Co., China). Then rats were killed and the aortas were immediately removed for study.

### Cell culture

Rat VSMCs were isolated from the thoracic aorta of SD rats (120-150 g) [7]. Briefly, after partial removal of external connective tissues, rat thoracic aortas were cut into small pieces (about 2-3 mm each), placed in Dulbecco’s modified Eagle’s medium (DMEM) containing 20% fetal bovine serum (FBS), 100 U/ml penicillin and 100 μg/ml streptomycin, and incubated at 37 ^o^C in an incubator containing 95% air and 5% CO_2_. VSMCs migrating from explants were collected and maintained in DMEM containing 10% FBS. Young (passage 4-6) and senescent (passage 14-18) VSMCs were used for experiments [3].

Mice VSMCs were isolated from 8-week old IMD^SMC-/-^ mice or WT littermate control mice as described [9] with minor modification. Briefly, five thoracic aortas were digested with 1 mg/mL type II collagenase (#LS004176; Worthington) at 37 °C for 10-15 min. The adventitia and endothelium were removed. Aortas were placed in DMEM containing 10% FBS, 100 U/ml penicillin and 100 μg/ml streptomycin overnight in a 37 °C incubator with 5% CO_2_, then digested in 5 mL of type II collagenase (1 mg/mL) and elastase I (0.25 mg/mL) (#E1250; Sigma-Aldrich, St. Louis, MO) for 90 min at 37 °C. The cellular digests were centrifuged at 1,000 rpm for 3 min, and cells were cultured in DMEM containing 20% FBS in culture dishes coated with gelatin (#G7041; Sigma-Aldrich) at 37 °C, 5% CO_2_. VSMCs were verified by α-SMA staining. Young VSMCs (passage 5-6) were used for experiments.

Human VSMC CRL1999 cells were from American Type Culture Collection (ATCC, Manassas, VA, USA) and were cultured in DMEM containing 10% FBS, 100 U/ml penicillin and 100 μg/ml streptomycin. Young (passage 4-6) and senescent (passage 14-18) VSMCs were used for experiments.

For calcification, confluent VSMCs were incubated in DMEM containing 10% FBS, 100 U/ml penicillin and 100 μg/ml streptomycin, added with 0.7 mmol/L CaCL_2_ (DMEM containing 1.8 mmol/L CaCl_2_) and 5 mmol/L β-glycerophosphate, and cultured at 37 ^o^C in an incubator containing 95% air and 5% CO_2_ for 12 days. The medium was changed every 2 to 3 days, and CaCl_2_ and β-glycerophosphate were also re-added. The control VSMCs were cultured in DMEM containing 10% FBS, 100 U/ml penicillin and 100 μg/ml streptomycin, but without CaCl_2_ and β-glycerophosphate, and the medium was also changed every 2 to 3 days [7].

To explored the role of CRLR/RAMPs in mediating the effect of IMD on sirt1, rat senescent VSMCs were preincubated with IMD_17-47_ (Phoenix Pharmaceuticals, Belmont, CA, USA), an effective antagonist of IMD receptor complex CRLR/RAMPs, with a concentration of 10^-6^ mol/L for 30 min [26, 50]. Then the cells were treated with IMD_1-53_ with a concentration of 10^-7^ mol/L for another 30 min, and induced calcification by using CaCL_2_ and β-glycerophosphate. The medium was changed every 2 to 3 days, and the steps were repeated each time [7]. After treatment for 12 days, the cells were collected and studied.

To investigate the signaling pathway by which IMD_1-53_ regulates senescence-associated VSMC calcification, rat senescent VSMCs were preincubated with the PI3K inhibitor LY294002 (10 μmol/L), AMPK inhibitor Compound C (10 μmol/L) or PKA inhibitor H89 (10 μmol/L) for 30 min. Then the cells were treated with IMD_1-53_ (10^-7^ mol/L) for another 30 min, and induced calcification by using CaCL_2_ and β-glycerophosphate. The medium was changed every 2 to 3 days, and the steps were repeated each time [7]. After treatment for 12 days, the cells were collected and studied.

### Hematoxylin and eosin (H&E) staining

Segments of rat thoracic aortas were placed in 4% phosphate buffered neutral formalin for 8 h, then immersed in 20% sucrose solution for storage; aorta samples were dehydrated and embedded in paraffin, cut into 5-μm-thick sections, then underwent H&E staining as we described previously [7].

### Alizarin red staining and von Kossa staining

For Alizarin red staining, cultured VSMCs were washed with phosphate-buffered saline (PBS) for three times (3 min each), and then were fixed in 4% phosphate buffered neutral formalin for 15 min. The cells washed in distilled water and exposed to Alizarin red staining solution (pH 4.2, 1%) for 30 min, then washed again with distilled water and observed by microscopy. To examine aorta calcification, slides were dehydrated, rinsed rapidly in distilled water, and placed in Alizarin red staining solution for 5 min, then tissues were photographed [7].

For von Kossa staining, sections were dehydrated before being immersed in 1% silver nitrate solution for 1 h under an intense sunbeam, then in 5% sodium thiosulfate for 2 min, counterstained with aldehyde-fuchsin, then observed by microscopy [7].

### Sirius red staining

Collagen was quantified by Sirius red staining as described previously [51]. The arterial media was delineated and the percentage of media positive for red staining was assessed by using Image-Pro Plus v6.0 (Media Cybernetics, Rockville, MD, USA).

### Senescence-associated β-galactosidase (SA-β-gal) staining

SA-β-gal staining involved using a commercial kit (#9860S; Cell Signaling Technology, Danvers, MA, USA). Thoracic aorta sections or VSMCs were fixed in 4% paraformaldehyde for 20 min, rinsed with PBS and incubated with β-galactosidase staining solution at 37 °C overnight, then photographed. Staining data were quantified by using Image-Pro Plus v6.0.

### Immunostaining

Sections (7 μm) of rat thoracic aortas freshly embedded in OCT underwent immunofluorescence staining. Sections were first fixed in 4% paraformaldehyde for 15 min and permeabilized with 0.1% Triton X-100 for 10 min. Non-specific binding was reduced by incubating slides in 10% goat sera diluted in PBS for 60 min at 37 °C. Sections were incubated with antibodies against sirt1 (#ab110304, 1:100; Abcam, Cambridge, MA, USA) or α-SMA (#ab5694, 1:100; Abcam) at 4 °C overnight, then rinsed with PBS and incubated with fluorescein-labeled secondary antibodies. Nuclei were stained with Hoechst 33342 (Sigma-Aldrich). Images were acquired under a Leica fluorescence microscopy (Leica Imaging Systems, Cambridge, UK).

Sections (5 μm) of rat thoracic aortas embedded in paraffin underwent immunohistochemical staining. Sections were incubated with antibodies against IMD (#sc-86272, 1:50; Santa Cruz Biotechnology, Santa Cruz, CA), RUNX2 (#ab23981, 1:100; Abcam) or p21 (#ab109199, 1:100; Abcam), then with secondary antibody for 1 h at 37 °C. Nuclei were stained with hematoxylin, then treated with DAB. Immunostaining data were quantified by using Image-Pro Plus v6.0.

### Quantification of calcium content and ALP activity assay

Calcium content was determined as described [7]. In brief, aortic segments without adventitia were dried at 60 °C and weighed. Then, tissues were dissolved in HNO_3_ and dried at 180 °C in an oven overnight and re-dissolved with the blank solution (27 nmol/L KCl, 27 μmol/L LaCl_3_ in deionized water). VSMCs were washed with PBS and decalcified with 0.6 mol/L HCl. Calcium content was analyzed by using a commercial kit (#340; Biosino Bio-Technology and Science, Beijing). Data were normalized to aortic dry weight or total protein level in VSMCs.

Aortic tissue samples and VSMC lysates were prepared as described [7]. ALP activity was measured by using an ALP assay kit (#A059-1; Nanjing Jiancheng Bioengineering, Nanjing, China). Results were normalized to level of total protein.

### Western blot analysis

Aortic tissues or cell extracts containing equal amounts of total protein were resolved by 10% or 12% SDS-PAGE, then transferred to a nitrocellulose membrane. Nonspecific proteins were blocked with 5% nonfat dried milk for 1 h, then incubated with the primary antibodies for β-actin (#sc-47778; 1:3000), GAPDH (#sc-47724; 1:1000; both Santa Cruz Biotechnology), CRLR (#GTX64616; 1:300; GeneTex, Irvine, CA, USA), RAMP1 (#ab156575; 1:1000; Abcam), RAMP2 (#sc-365240; 1:100), RAMP3 (#sc-365313; 1:100; both Santa Cruz Biotechnology); sirt1 (#ab110304; 1:200), klotho (#ab203576; 1:500), RUNX2 (#ab23981; 1:1000), BMP2 (#ab14933; 1:500; all Abcam); p53 (#sc-6243; 1:100; Santa Cruz Biotechnology), and acetyl-p53 (#ab183544; 1:500), p21 (#ab109199; 1:500), p16 (#ab189034; 1:500), α-SMA (#ab5694; 1:3000), SM-22α (#ab10135; 1:1000; all Abcam); MGP (#10734-1-AP; 1:500; Proteintech, USA), AMPK (#2532; 1:1000), p-AMPK (Thr172) (#2531; 1:1000), Akt (#9272; 1:1000), p-Akt (Ser473) (#4060; 1:1000), PKA (#4782; 1:1000) or p-PKA (Thr197) (#4781; 1:1000; all Cell Signaling Technology) overnight at 4 °C, then secondary antibody (horseradish peroxidase-conjugated anti-rabbit, anti-mouse or anti-goat IgG) (Santa Cruz Biotechnology) for 1 h. The proteins were detected by enhanced chemiluminescence. Protein expression was analyzed by using ImageJ and normalized to β-actin or GAPDH expression.

### Real-time (RT) PCR analysis

Total RNA was isolated and reverse transcribed. RT-PCR amplification involved using the Applied Biosystems 7500 fast PCR System (Life Technologies, USA) and SYBR Green I reagent (#FP205-02; Tiangen Biotech; Beijing). The cycle threshold (Ct) was determined as the number of PCR cycles required for a given reaction to reach an arbitrary fluorescence value within the linear amplification range. Relative quantification was performed according to the 2^-ΔΔCt^ method, with GAPDH level as a reference. The forward and reverse PCR primers are in Supplementary Table 1.

### siRNA transfection

Rat senescent VSMCs at 50% confluence were incubated with sirt1 siRNA or negative control siRNA at a final concentration of 20 nmol by using Lipofectamine RNAiMAX Transfection Reagent (Invitrogen, Carlsbad, CA, USA) for 6 h. To evaluate the efficiency of target gene knockdown, cells underwent RT-PCR and western blot analysis at 36 and 72 h after transfection, respectively. After 6 h of transfection with serum-free DMEM, the serum-containing medium was replaced. At 48 h after transfection, cells were treated with IMD_1-53_ and calcification was induced for 7 days. siRNA transfection procedures were performed every 48 h.

### Statistical analysis

All data are expressed as mean ± SD. Statistical analysis involved use of Graphpad Prism v5.00 for Windows (GraphPad Software Inc., San Diego, CA, USA). Student *t* test was used to compare two groups and one-way ANOVA followed by Student-Newman-Keuls test for more than two groups. Statistical significance was accepted at *P*<0.05.

## Supplementary Material

Supplementary Figures

Supplementary Tables

## References

[1] Nakano-Kurimoto R, Ikeda K, Uraoka M, Nakagawa Y, Yutaka K, Koide M, Takahashi T, Matoba S, Yamada H, Okigaki M, Matsubara H. Replicative senescence of vascular smooth muscle cells enhances the calcification through initiating the osteoblastic transition. Am J Physiol Heart Circ Physiol. 2009; 297:H1673–84. 10.1152/ajpheart.00455.2009 19749165

[2] Sanchis P, Ho CY, Liu Y, Beltran LE, Ahmad S, Jacob AP, Furmanik M, Laycock J, Long DA, Shroff R, Shanahan CM. Arterial “inflammaging” drives vascular calcification in children on dialysis. Kidney Int. 2019; 95:958–72. 10.1016/j.kint.2018.12.014 30827513 PMC6684370

[3] Liu Y, Drozdov I, Shroff R, Beltran LE, Shanahan CM. Prelamin A accelerates vascular calcification via activation of the DNA damage response and senescence-associated secretory phenotype in vascular smooth muscle cells. Circ Res. 2013; 112:e99–109. 10.1161/CIRCRESAHA.111.300543 23564641

[4] Pescatore LA, Gamarra LF, Liberman M. Multifaceted mechanisms of vascular calcification in aging. Arterioscler Thromb Vasc Biol. 2019; 39:1307–16. 10.1161/ATVBAHA.118.311576 31144990

[5] Alique M, Ruíz-Torres MP, Bodega G, Noci MV, Troyano N, Bohórquez L, Luna C, Luque R, Carmona A, Carracedo J, Ramírez R. Microvesicles from the plasma of elderly subjects and from senescent endothelial cells promote vascular calcification. Aging (Albany NY). 2017; 9:778–89. 10.18632/aging.101191 28278131 PMC5391231

[6] Cai Y, Xu MJ, Teng X, Zhou YB, Chen L, Zhu Y, Wang X, Tang CS, Qi YF. Intermedin inhibits vascular calcification by increasing the level of matrix gamma-carboxyglutamic acid protein. Cardiovasc Res. 2010; 85:864–73. 10.1093/cvr/cvp366 19910445

[7] Chang JR, Guo J, Wang Y, Hou YL, Lu WW, Zhang JS, Yu YR, Xu MJ, Liu XY, Wang XJ, Guan YF, Zhu Y, Du J, et al. Intermedin1-53 attenuates vascular calcification in rats with chronic kidney disease by upregulation of α-Klotho. Kidney Int. 2016; 89:586–600. 10.1016/j.kint.2015.12.029 26880455

[8] Takemura A, Iijima K, Ota H, Son BK, Ito Y, Ogawa S, Eto M, Akishita M, Ouchi Y. Sirtuin 1 retards hyperphosphatemia-induced calcification of vascular smooth muscle cells. Arterioscler Thromb Vasc Biol. 2011; 31:2054–62. 10.1161/ATVBAHA.110.216739 21719763

[9] Badi I, Mancinelli L, Polizzotto A, Ferri D, Zeni F, Burba I, Milano G, Brambilla F, Saccu C, Bianchi ME, Pompilio G, Capogrossi MC, Raucci A. miR-34a promotes vascular smooth muscle cell calcification by downregulating SIRT1 (Sirtuin 1) and Axl (AXL receptor tyrosine kinase). Arterioscler Thromb Vasc Biol. 2018; 38:2079–90. 10.1161/ATVBAHA.118.311298 30026277

[10] Stenvinkel P, Luttropp K, McGuinness D, Witasp A, Qureshi AR, Wernerson A, Nordfors L, Schalling M, Ripsweden J, Wennberg L, Söderberg M, Bárány P, Olauson H, Shiels PG. *CDKN2A/p16INK4^a^* expression is associated with vascular progeria in chronic kidney disease. Aging (Albany NY). 2017; 9:494–507. 10.18632/aging.101173 28192277 PMC5361677

[11] Kida Y, Goligorsky MS. Sirtuins, cell Senescence, and vascular aging. Can J Cardiol. 2016; 32:634–41. 10.1016/j.cjca.2015.11.022 26948035 PMC4848124

[12] Imai S, Armstrong CM, Kaeberlein M, Guarente L. Transcriptional silencing and longevity protein Sir2 is an NAD-dependent histone deacetylase. Nature. 2000; 403:795–800. 10.1038/35001622 10693811

[13] Satoh A, Brace CS, Rensing N, Cliften P, Wozniak DF, Herzog ED, Yamada KA, Imai S. Sirt1 extends life span and delays aging in mice through the regulation of Nk2 homeobox 1 in the DMH and LH. Cell Metab. 2013; 18:416–30. 10.1016/j.cmet.2013.07.013 24011076 PMC3794712

[14] Ren J, Yang L, Zhu L, Xu X, Ceylan AF, Guo W, Yang J, Zhang Y. Akt2 ablation prolongs life span and improves myocardial contractile function with adaptive cardiac remodeling: role of Sirt1-mediated autophagy regulation. Aging Cell. 2017; 16:976–87. 10.1111/acel.12616 28681509 PMC5595687

[15] Ying Y, Jiang C, Zhang M, Jin J, Ge S, Wang X. Phloretin protects against cardiac damage and remodeling via restoring SIRT1 and anti-inflammatory effects in the streptozotocin-induced diabetic mouse model. Aging (Albany NY). 2019; 11:2822–35. 10.18632/aging.101954 31076562 PMC6535073

[16] Feng T, Liu P, Wang X, Luo J, Zuo X, Jiang X, Liu C, Li Y, Li N, Chen M, Zhu N, Han X, Liu C, et al. SIRT1 activator E1231 protects from experimental atherosclerosis and lowers plasma cholesterol and triglycerides by enhancing ABCA1 expression. Atherosclerosis. 2018; 274:172–81. 10.1016/j.atherosclerosis.2018.04.039 29787963

[17] Chen HZ, Wang F, Gao P, Pei JF, Liu Y, Xu TT, Tang X, Fu WY, Lu J, Yan YF, Wang XM, Han L, Zhang ZQ, et al. Age-associated sirtuin 1 reduction in vascular smooth muscle links vascular senescence and inflammation to abdominal aortic aneurysm. Circ Res. 2016; 119:1076–88. 10.1161/CIRCRESAHA.116.308895 27650558 PMC6546422

[18] Akiyoshi T, Ota H, Iijima K, Son BK, Kahyo T, Setou M, Ogawa S, Ouchi Y, Akishita M. A novel organ culture model of aorta for vascular calcification. Atherosclerosis. 2016; 244:51–58. 10.1016/j.atherosclerosis.2015.11.005 26584139

[19] Zhou YB, Gao Q, Li P, Han Y, Zhang F, Qi YF, Tang CS, Gao XY, Zhu GQ. Adrenomedullin attenuates vascular calcification in fructose-induced insulin resistance rats. Acta Physiol (Oxf). 2013; 207:437–46. 10.1111/apha.12033 23121999

[20] Fujitsuka N, Asakawa A, Morinaga A, Amitani MS, Amitani H, Katsuura G, Sawada Y, Sudo Y, Uezono Y, Mochiki E, Sakata I, Sakai T, Hanazaki K, et al. Increased ghrelin signaling prolongs survival in mouse models of human aging through activation of sirtuin1. Mol Psychiatry. 2016; 21:1613–23. 10.1038/mp.2015.220 26830139 PMC5078860

[21] Roh J, Chang CL, Bhalla A, Klein C, Hsu SY. Intermedin is a calcitonin/calcitonin gene-related peptide family peptide acting through the calcitonin receptor-like receptor/receptor activity-modifying protein receptor complexes. J Biol Chem. 2004; 279:7264–74. 10.1074/jbc.M305332200 14615490

[22] Kobayashi Y, Liu YJ, Gonda T, Takei Y. Coronary vasodilatory response to a novel peptide, adrenomedullin 2. Clin Exp Pharmacol Physiol. 2004 (Suppl 2); 31:S49–50. 10.1111/j.1440-1681.2004.04115.x 15649289

[23] Yang JH, Jia YX, Pan CS, Zhao J, Ouyang M, Yang J, Chang JK, Tang CS, Qi YF. Effects of intermedin(1-53) on cardiac function and ischemia/reperfusion injury in isolated rat hearts. Biochem Biophys Res Commun. 2005; 327:713–19. 10.1016/j.bbrc.2004.12.071 15649405

[24] Ni X, Zhang J, Tang C, Qi Y. Intermedin/adrenomedullin2: an autocrine/paracrine factor in vascular homeostasis and disease. Sci China Life Sci. 2014; 57:781–89. 10.1007/s11427-014-4701-7 25104450

[25] Yang JH, Pan CS, Jia YX, Zhang J, Zhao J, Pang YZ, Yang J, Tang CS, Qi YF. Intermedin1-53 activates L-arginine/nitric oxide synthase/nitric oxide pathway in rat aortas. Biochem Biophys Res Commun. 2006; 341:567–72. 10.1016/j.bbrc.2006.01.010 16434024

[26] Lu WW, Jia LX, Ni XQ, Zhao L, Chang JR, Zhang JS, Hou YL, Zhu Y, Guan YF, Yu YR, Du J, Tang CS, Qi YF. Intermedin1-53 attenuates abdominal aortic aneurysm by inhibiting oxidative stress. Arterioscler Thromb Vasc Biol. 2016; 36:2176–90. 10.1161/ATVBAHA.116.307825 27634835

[27] Yang R, Fang W, Liang J, Lin C, Wu S, Yan S, Hu C, Ke X. Apelin/APJ axis improves angiotensin II-induced endothelial cell senescence through AMPK/SIRT1 signaling pathway. Arch Med Sci. 2018; 14:725–34. 10.5114/aoms.2017.70340 30002688 PMC6040122

[28] Gao D, Zuo Z, Tian J, Ali Q, Lin Y, Lei H, Sun Z. Activation of SIRT1 attenuates klotho deficiency-induced arterial stiffness and hypertension by enhancing AMP-activated protein kinase activity. Hypertension. 2016; 68:1191–99. 10.1161/HYPERTENSIONAHA.116.07709 27620389 PMC5063709

[29] Carloni S, Riparini G, Buonocore G, Balduini W. Rapid modulation of the silent information regulator 1 by melatonin after hypoxia-ischemia in the neonatal rat brain. J Pineal Res. 2017; 63:e12434. 10.1111/jpi.12434 28708259

[30] Niederhoffer N, Bobryshev YV, Lartaud-Idjouadiene I, Giummelly P, Atkinson J. Aortic calcification produced by vitamin D3 plus nicotine. J Vasc Res. 1997; 34:386–98. 10.1159/000159247 9349732

[31] Kieffer P, Robert A, Capdeville-Atkinson C, Atkinson J, Lartaud-Idjouadiene I. Age-related arterial calcification in rats. Life Sci. 2000; 66:2371–81. 10.1016/S0024-3205(00)00567-1 10864099

[32] Jegger D, da Silva RF, Lartaud I, Gaillard V, Jeanrenaud X, Nasratullah M, von Segesser LK, Atkinson J, Segers P, Tevaearai H, Stergiopulos N. Effects of an aging vascular model on healthy and diseased hearts. Am J Physiol Heart Circ Physiol. 2007; 293:H1334–43. 10.1152/ajpheart.00341.2007 17616750

[33] Lv Z, Wu K, Chen X, Zhang X, Hong B. Plasma intermedin levels in patients with acute myocardial infarction. Peptides. 2013; 43:121–25. 10.1016/j.peptides.2013.03.007 23499766

[34] Qin YW, Teng X, He JQ, Du J, Tang CS, Qi YF. Increased plasma levels of intermedin and brain natriuretic peptide associated with severity of coronary stenosis in acute coronary syndrome. Peptides. 2013; 42:84–88. 10.1016/j.peptides.2013.01.011 23391507

[35] Bardeesi AS, Gao J, Zhang K, Yu S, Wei M, Liu P, Huang H. A novel role of cellular interactions in vascular calcification. J Transl Med. 2017; 15:95. 10.1186/s12967-017-1190-z 28464904 PMC5414234

[36] Shanahan CM, Crouthamel MH, Kapustin A, Giachelli CM. Arterial calcification in chronic kidney disease: key roles for calcium and phosphate. Circ Res. 2011; 109:697–711. 10.1161/CIRCRESAHA.110.234914 21885837 PMC3249146

[37] López-Otín C, Blasco MA, Partridge L, Serrano M, Kroemer G. The hallmarks of aging. Cell. 2013; 153:1194–217. 10.1016/j.cell.2013.05.039 23746838 PMC3836174

[38] Childs BG, Durik M, Baker DJ, van Deursen JM. Cellular senescence in aging and age-related disease: from mechanisms to therapy. Nat Med. 2015; 21:1424–35. 10.1038/nm.4000 26646499 PMC4748967

[39] Burton DG, Giles PJ, Sheerin AN, Smith SK, Lawton JJ, Ostler EL, Rhys-Williams W, Kipling D, Faragher RG. Microarray analysis of senescent vascular smooth muscle cells: A link to atherosclerosis and vascular calcification. Exp Gerontol. 2009; 44:659–65. 10.1016/j.exger.2009.07.004 19631729

[40] Kitada M, Ogura Y, Koya D. The protective role of Sirt1 in vascular tissue: its relationship to vascular aging and atherosclerosis. Aging (Albany NY). 2016; 8:2290–307. 10.18632/aging.101068 27744418 PMC5115889

[41] Ong AL, Ramasamy TS. Role of Sirtuin1-p53 regulatory axis in aging, cancer and cellular reprogramming. Ageing Res Rev. 2018; 43:64–80. 10.1016/j.arr.2018.02.004 29476819

[42] Teng X, Song J, Zhang G, Cai Y, Yuan F, Du J, Tang C, Qi Y. Inhibition of endoplasmic reticulum stress by intermedin(1-53) protects against myocardial injury through a PI3 kinase-Akt signaling pathway. J Mol Med (Berl). 2011; 89:1195–205. 10.1007/s00109-011-0808-5 21909975

[43] Ni XQ, Lu WW, Zhang JS, Zhu Q, Ren JL, Yu YR, Liu XY, Wang XJ, Han M, Jing Q, Du J, Tang CS, Qi YF. Inhibition of endoplasmic reticulum stress by intermedin1-53 attenuates angiotensin II-induced abdominal aortic aneurysm in ApoE KO Mice. Endocrine. 2018; 62:90–106. 10.1007/s12020-018-1657-6 29943223

[44] Shen J, Zhang N, Lin YN, Xiang P, Liu XB, Shan PF, Hu XY, Zhu W, Tang YL, Webster KA, Cai R, Schally AV, Wang J, Yu H. Regulation of vascular calcification by growth hormone-releasing hormone and its agonists. Circ Res. 2018; 122:1395–408. 10.1161/CIRCRESAHA.117.312418 29618597 PMC5948169

[45] Zhang JS, Hou YL, Lu WW, Ni XQ, Lin F, Yu YR, Tang CS, Qi YF. Intermedin1-53 protects against myocardial fibrosis by inhibiting endoplasmic reticulum stress and inflammation induced by homocysteine in apolipoprotein E-deficient mice. J Atheroscler Thromb. 2016; 23:1294–306. 10.5551/jat.34082 27052784 PMC5113747

[46] Wang H, Dong X, Liu Z, Zhu S, Liu H, Fan W, Hu Y, Hu T, Yu Y, Li Y, Liu T, Xie C, Gao Q, et al. Resveratrol suppresses rotenone-induced neurotoxicity through activation of SIRT1/Akt1 signaling pathway. Anat Rec (Hoboken). 2018; 301:1115–25. 10.1002/ar.23781 29350822

[47] Li Z, Han X. Resveratrol alleviates early brain injury following subarachnoid hemorrhage: possible involvement of the AMPK/SIRT1/autophagy signaling pathway. Biol Chem. 2018; 399:1339–50. 10.1515/hsz-2018-0269 30067508

[48] Chen ML, Yi L, Jin X, Liang XY, Zhou Y, Zhang T, Xie Q, Zhou X, Chang H, Fu YJ, Zhu JD, Zhang QY, Mi MT. Resveratrol attenuates vascular endothelial inflammation by inducing autophagy through the cAMP signaling pathway. Autophagy. 2013; 9:2033–45. 10.4161/auto.26336 24145604

[49] Duan XH, Chang JR, Zhang J, Zhang BH, Li YL, Teng X, Zhu Y, Du J, Tang CS, Qi YF. Activating transcription factor 4 is involved in endoplasmic reticulum stress-mediated apoptosis contributing to vascular calcification. Apoptosis. 2013; 18:1132–44. 10.1007/s10495-013-0861-3 23686245

[50] Kandilci HB, Gumusel B, Lippton H. Intermedin/adrenomedullin-2 (IMD/AM2) relaxes rat main pulmonary arterial rings via cGMP-dependent pathway: role of nitric oxide and large conductance calcium-activated potassium channels (BK(Ca)). Peptides. 2008; 29:1321–28. 10.1016/j.peptides.2008.04.008 18538894

[51] Donato AJ, Walker AE, Magerko KA, Bramwell RC, Black AD, Henson GD, Lawson BR, Lesniewski LA, Seals DR. Life-long caloric restriction reduces oxidative stress and preserves nitric oxide bioavailability and function in arteries of old mice. Aging Cell. 2013; 12:772–83. 10.1111/acel.12103 23714110 PMC3772986

