# Supplementary material for: Intermedin1-53 attenuates aging-associated vascular calcification in rats by upregulating sirtuin 1
Source: Aging (Albany NY). 2020 Mar 31;12(7):5651–74. doi: 10.18632/aging.102934 (PMC7185112; doi:10.18632/aging.102934)
Supplement: Supplementary Figures [file aging-12-102934-s002..pdf]

SUPPLEMENTARY FIGURES

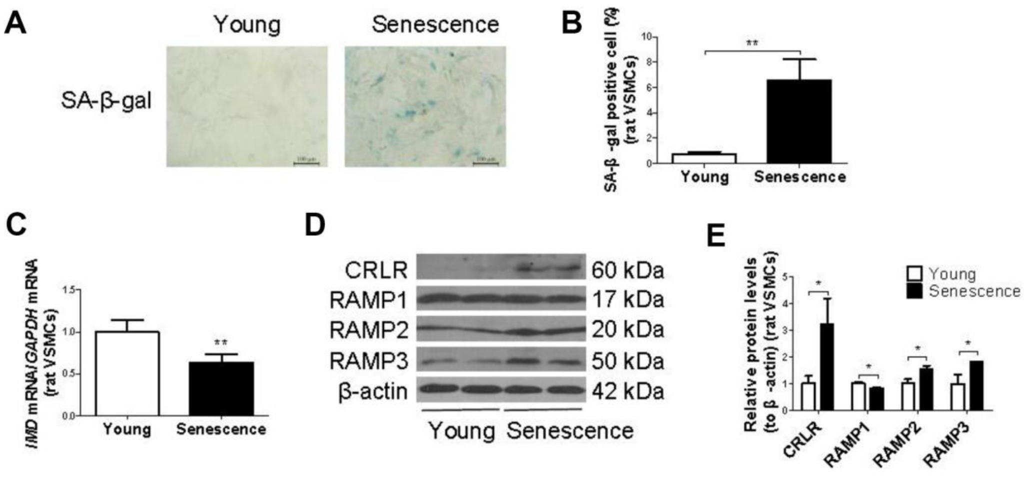

Supplementary Figure 1. The level of *IMD* and its receptors in rat young (passage 4-6) and senescent (passage 14-18) VSMCs. (**A**) SA-β-gal staining (Scale bar=100 μm) and (**B**) quantification of β-galactosidase-positive staining (blue) in rat young and senescent VSMCs (n=6). (**C**) RT-PCR analysis of mRNA level of *IMD* in rat VSMCs (n=3). (**D**) Western blot analysis of protein levels of calcitonin receptor-like receptor (CRLR), receptor activity-modifying protein 1 (RAMP1), 2 and 3 in rat VSMCs, and (**E**) quantification (n=3). Data are mean ± SD. **P*<0.05, ***P*<0.01.

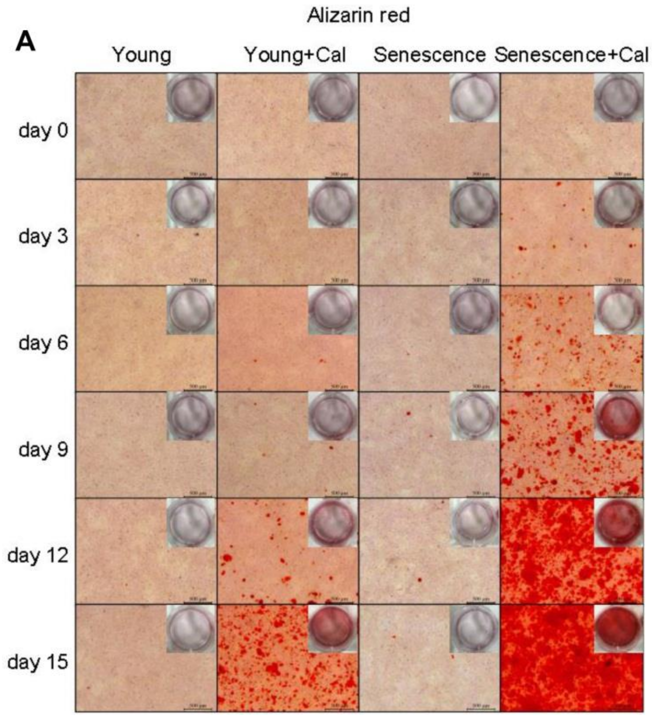

Supplementary Figure 2. Calcification in rat young (passage 4-6) and senescent (passage 14-18) VSMCs. (**A**) Alizarin red staining for calcium deposition (positive staining: red) with calcification time in rat VSMCs (Scale bar=500 μm).

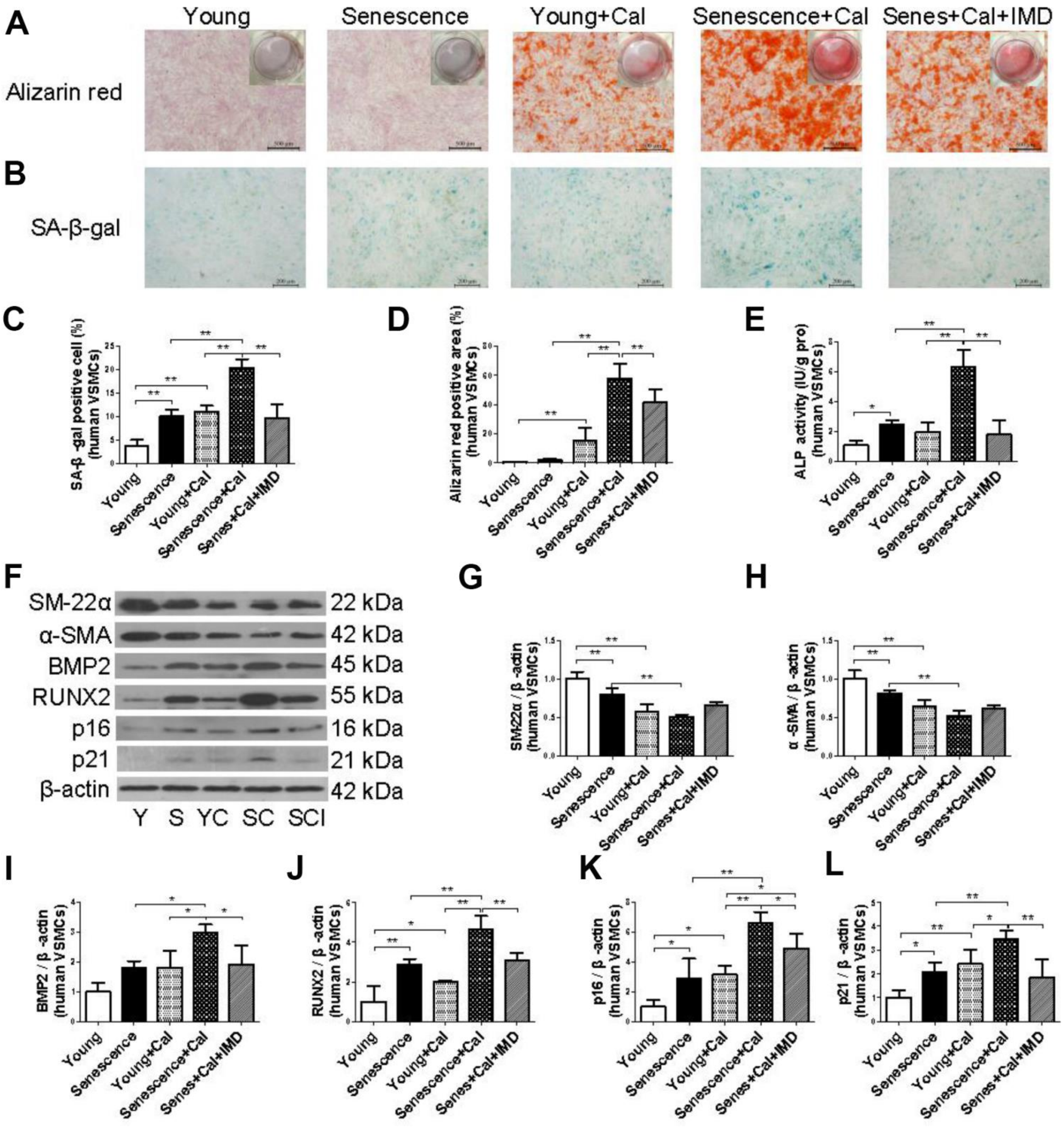

Supplementary Figure 3. Exogenous IMD$_{1-53}$ attenuated senescence-associated calcification in human VSMCs. (**A**) Alizarin red staining for human VSMCs (positive staining: red) (Scale bar=500 μm). (**B**) SA-β-gal staining (blue) (Scale bar=100 μm), and (**C**) quantification of β-galactosidase-positive staining (n=6) and (**D**) calcium deposition-positive staining (n=6) in human VSMCs. (**E**) ALP activity assay (n=6) in human VSMCs. (**F**) Western blot analysis of protein levels of smooth muscle 22 alpha (SM-22α), alpha smooth muscle actin (α-SMA), bone morphogenetic protein 2 (BMP2), runt-related transcription factor 2 (RUNX2), and cyclin-dependent kinase inhibitors p16 and p21 in human VSMCs, and (**G**–**L**) quantification (n=3). Y=young. S=senescence. YC=young+calcification. SC=senescence+calcification. SCI=senescence+calcification+IMD$_{1-53}$. Data are mean ± SD. **P*<0.05, ***P*<0.01.

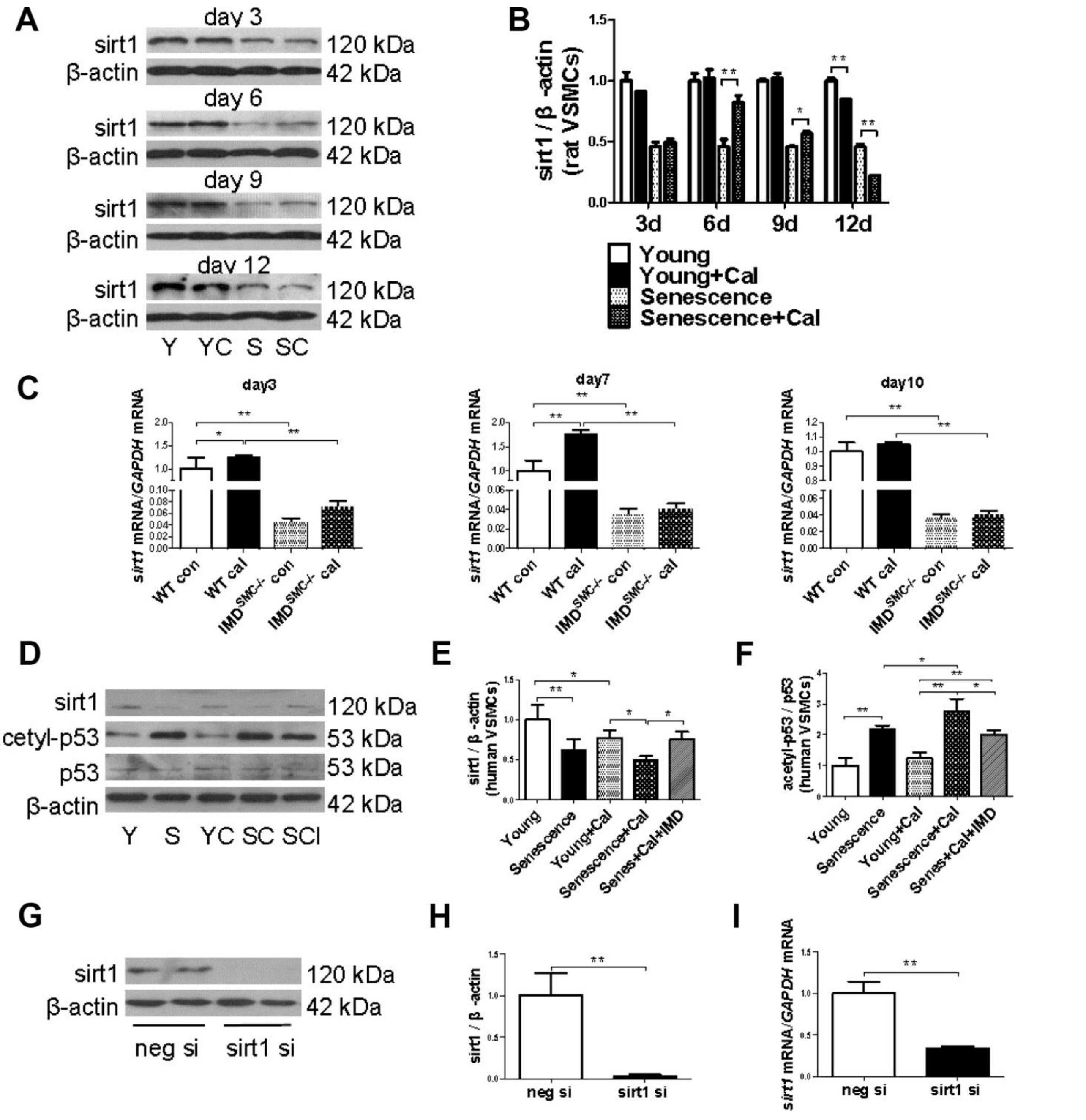

Supplementary Figure 4. IMD$_{1-53}$ inhibited aging-associated vascular calcification by increasing sirt1 expression and deacetylase activity. (**A**) Western blot analysis of protein level of sirt1 with calcification time in rat VSMCs, and (**B**) quantification (n=3). (**C**) RT-PCR analysis of mRNA level of *sirt1* with calcification time in WT and IMD$^{SMC-/-}$ mouse VSMCs (passage 5-6) (n=3). (**D**) Western blot analysis of protein levels of sirt1, acetylation p53 (acetyl-p53), and total p53 (p53) in human VSMCs, and (**E**–**F**) quantification (n=3). (**G**–**I**) Western blot and quantitative RT-PCR analysis of protein and mRNA levels of sirt1 after 72 h and 36 h, respectively, with siRNA addition (n=3). WT=wild type. IMD$^{SMC-/-}$=VSMC-specific *IMD*-deficient. Con=control. Cal=calcification. Y=young. S=senescence. YC=young+calcification. SC=senescence+calcification. SCI=senescence+calcification+IMD$_{1-53}$. neg si=negative siRNA. sirt1 si=sirt1 siRNA. Data are mean ± SD. *P<0.05, **P<0.01.

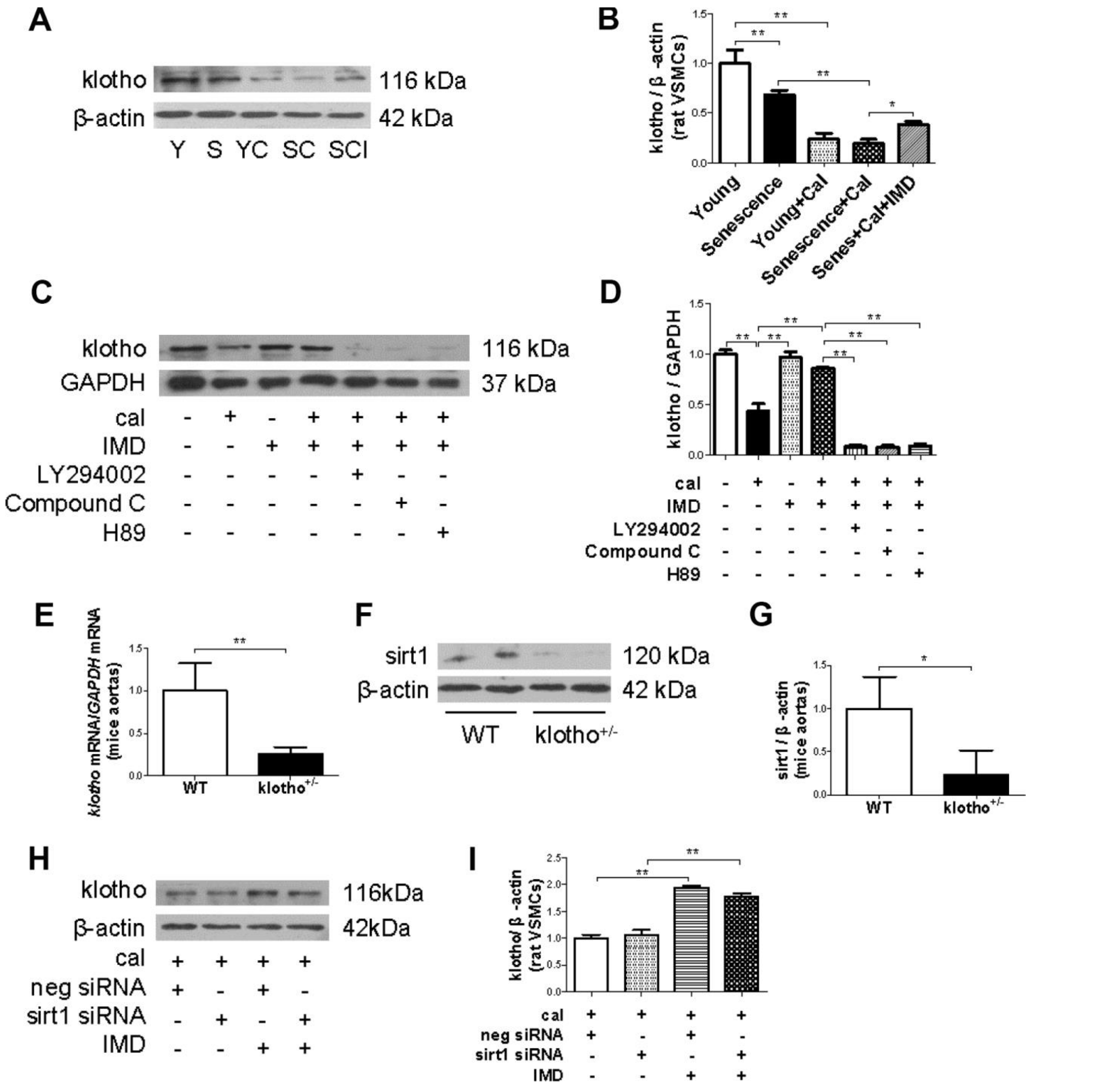

Supplementary Figure 5. The klotho-sirt1 axis in IMD$_{1-53}$ attenuating senescence-associated VSMC calcification. (**A**) Western blot analysis of protein level of klotho in rat VSMCs, and (**B**) quantification (n=3). (**C**) Western blot analysis of protein level of klotho in rat senescent VSMCs preincubation with or without PI3K inhibitor LY294002, AMPK inhibitor Compound C or PKA inhibitor H89 (all 10 μmol/L) before IMD$_{1–53}$ administration and calcification induction, and (**D**) quantification (n=3). (**E**) RT-PCR analysis of mRNA level of *klotho* in aortas from WT and klotho$^{+/-}$ mouse aortas (n=3). (**F**) Western blot analysis of protein level of sirt1 in WT and klotho$^{+/-}$ mice, and (**G**) quantification (n=4). (**H**) Western blot analysis of protein level of klotho in calcified-rat senescent VSMCs treated with IMD$_{1-53}$ plus sirt1 siRNA or negative siRNA, and (**I**) quantification (n=3). WT=wild type. klotho$^{+/-}$= heterozygous *klotho*-deficient. Cal=calcification. Y=young. S=senescence. YC=young+calcification. SC=senescence+calcification. SCI=senescence+calcification+IMD$_{1-53}$. neg si=negative siRNA. sirt1 si=sirt1 siRNA. Data are mean ± SD. **P*<0.05, ***P*<0.01.
