# Supplementary material for: Intermedin1-53 attenuates aging-associated vascular calcification in rats by upregulating sirtuin 1
Source: Aging (Albany NY). 2020 Mar 31;12(7):5651–74. doi: 10.18632/aging.102934 (PMC7185112; doi:10.18632/aging.102934)
Supplement: Supplementary Tables [file aging-12-102934-s001..pdf]

SUPPLEMENTARY TABLES

Supplementary Table 1. The forward and reverse PCR primers.

Targets		Sequence	Annealing temperature (°C)
IMD (rat/mouse)	forward	5'- CTTGCCAGCTGTCTCCAGAT -3'	60
	reverse	5'- CAGGTAGAGGAGGCTGATGC -3'	
CRLR (rat)	forward	5'- AGAGCCTAAGTTGCCAACGGA -3'	60
	reverse	5'- CTTCTCCGCAAACACAGCCA -3'	
RAMP1 (rat)	forward	5'-CACCACCGCTACTTCAGCAA- 3'	60
	reverse	5'-GGCAGTCATGAGCAGTGTGAC- 3'	
RAMP2 (rat)	forward	5'-TGCTTGGAGTACGAGGCAGA- 3'	60
	reverse	5'-GAAGGTAGGCTGCACCAAGG- 3'	
RAMP3 (rat)	forward	5'-ACCTGTCGGAGTTCATCGTGT- 3'	60
	reverse	5'-TAGCCACGGTCAACAAGACTG- 3'	
sirt1 (rat/mouse)	forward	5'-CACATGCCAGAGTCCAAGTTT- 3'	60
	reverse	5'-GTCAGCTCCAGATCCTCCAG- 3'	
Klotho (mouse)	forward	5'- CCTCATGGATGGTTTCGAGT -3'	60
	reverse	5'- TTGGTAGAACAAGGCCGAAG -3'	
GAPDH (rat/mouse)	forward	5'-ACTTTGTCAAGCTCATTTCC- 3'	60
	reverse	5'-TGCAGCGAACTTTATTGATG- 3'	

Supplementary Table 2. Effect of IMD_{1-53} on hemodynamic parameters of old rats induced with vitamin D3 plus nicotine (VDN).

	Young	Young+VDN	Old	Old+VDN	Old+VDN+IMD
HR (beats/min)	395±39	427±48	342±55	393±25	377±34
SBP (mmHg)	103.5±8.25	129.71±11.55**	105.68±3.6	121.12±7.29#	113.02±10.07
DBP (mmHg)	63.73±4.25	89.19±14.26*	65.47±7.06	64.61±15.28	63.54±19.31
MBP (mmHg)	79.07±7.16	103.48±13.65*	79.01±5.63	83.26±11.53	80.03±15.88
LVSP (mmHg)	103.06±7.77	133.12±14.58**	107.52±8.61	127.73±9.56#	113.37±10.25§
LVEDP (mmHg)	-1.48±5.04	-2.92±7.89	-7.29±2.94	-4.42±1.29	-4.76±3.89
+dp/dtm (mmHg/s)	2899.24±581.68	3893.55±761.43	3512.88±674.57	3754.88±470.9	3649.29±226.38
-dp/dtm (mmHg/s)	-2430.63±584.96	-3411.58±743.92*	-2979.43±461.96	-3255.31±262.56	-2779.34±428.66

HR, heart rate; SBP, systolic blood pressure; DBP, diastolic blood pressure; MBP, mean blood pressure; LVSP, left ventricular systolic pressure; LVEDP, left ventricular end-diastolic pressure; ± dp/dtm, left ventricular peak rate of contraction and relaxation. Data are mean ± SD. n=5 in each group. *$P<0.05$, **$P<0.01$ *vs*. Young. #$P<0.05$ *vs*. Old. §$P<0.05$ *vs*. Old+VDN.
